# Large Language Models in Sensor-Driven Control Systems: Architectures, Challenges, and Opportunities

**DOI:** 10.3390/s26144350

**Published:** 2026-07-09

**Authors:** Fateme Aghaee, Hamid Reza Shaker

**Affiliations:** 1 SDU Centre for Industrial Mechanics, Institute of Mechanical and Electrical Engineering, University of Southern Denmark, 6400 Sønderborg, Denmark; aghaee@sdu.dk; 2 SDU Center for Energy Informatics, Maersk Mc-Kinney Moeller Institute, The Faculty of Engineering, University of Southern Denmark, 5230 Odense, Denmark

**Keywords:** large language models, sensor-driven control systems, hybrid architectures, robotics, industrial automation, cyber–physical systems, digital twins, supervisory control, human–AI interaction, trustworthy AI, evaluation and benchmarking

## Abstract

Large language models (LLMs) are increasingly being explored for integration into sensor-driven control systems across robotics, industrial automation, energy infrastructure, healthcare, smart environments, and other sensor-rich domains. This review synthesizes emerging research from the perspective of sensor-driven control systems, defined as systems in which sensing is substantively linked to monitoring, estimation, supervision, planning, decision-making, or actuation. Rather than treating LLMs as generic intelligent agents, the review examines their position within the sensing–decision–control chain and their interaction with state representations, supervisory logic, human operators, external tools, and classical control components. The paper develops a functional taxonomy of LLM roles based on proximity to actuation, grounding requirements, and deployment risk. This taxonomy reveals a clear maturity gradient: interpretive, supervisory, diagnostic, and engineering-support roles are currently the most credible and deployable, whereas runtime control participation remains the least mature and highest-risk form of integration. The analysis further shows that reliable implementations are predominantly hybrid. In such architectures, LLMs function as semantic and orchestration layers that augment, rather than replace, classical sensing, estimation, planning, and control. Key integration patterns include sensor-to-semantics pipelines, retrieval-augmented generation, tool use, agentic workflows, closed-loop refinement, and safety-aware mechanisms. Persistent challenges—including hallucination, weak physical grounding, latency, cybersecurity risks, and the lack of formal guarantees—highlight the need for rigorous operational evaluation and realistic benchmarks. The review concludes that LLMs are most credible as interpretive, supervisory, diagnostic, and human-facing intelligence layers embedded within hybrid architectures. Future progress will depend on deeper neuro-symbolic integration, efficient local deployment, human-centered autonomy, and stronger evaluation practices that preserve the strengths of classical control engineering while extending them with semantic reasoning and supervisory intelligence.

## 1. Introduction

### 1.1. Background and Motivation

Sensor-driven control systems tightly couple sensing, state estimation, decision-making, and actuation in closed-loop or supervisory operation. In such systems, sensors observe a plant, process, environment, or human user; estimation and interpretation modules convert raw measurements into usable state representations; decision layers determine appropriate actions or adjustments; and control and actuation components execute them in the physical world. This sensing–estimation–decision–actuation chain underpins a wide range of modern engineered systems, including autonomous robotics, industrial automation, cyber–physical systems (CPSs), smart energy infrastructures, connected buildings, and assistive healthcare technologies.

The demands placed on these systems are increasing as operational environments become more complex, data-rich, and interconnected. Robotics platforms increasingly combine vision, force/tactile, proprioceptive, and spatial sensing for navigation, manipulation, and human–robot interaction [1]. Industrial automation must integrate machine data, condition-monitoring signals, alarms, maintenance logs, and operator inputs across distributed CPSs [2,3]. Comparable challenges arise in energy systems, smart infrastructure, and healthcare, where reliable operation depends on interpreting heterogeneous, noisy, and multimodal sensor streams under real-time, safety, and resource constraints [4]. In this review, the term sensor-driven is therefore used broadly to include systems built on such imperfect inputs—including vision, light detection and ranging (LiDAR), telemetry, logs, alarms, and event traces—as well as the intermediate abstractions that connect these inputs to higher-level reasoning and operational decision-making.

LLMs have recently evolved into powerful general-purpose reasoning systems capable of semantic interpretation, contextual understanding, high-level planning, explanation generation, tool use, and natural human–machine interaction [5,6,7]. These capabilities make them promising additions to sensor-driven environments, particularly where low-level operational data must be connected to operator intent, procedural knowledge, domain expertise, and safety constraints. Rather than functioning only as conversational interfaces, LLMs are increasingly being explored as supervisory, interpretive, diagnostic, and coordination layers that help transform raw system complexity into actionable intelligence.

This convergence is especially timely because recent years have seen rapid growth in related research across previously siloed communities, including LLM-enabled industrial agents [8], multimodal robotic reasoning and control [9], cognitive layers for CPS [10], and digital-twin-assisted automation [11]. Yet the resulting literature remains fragmented across robotics, automation, autonomy, diagnostics, and human-centered operational AI, often without a shared vocabulary or a unified architectural perspective. Existing reviews tend to focus narrowly on robotics and embodied agents [12,13], generic LLM applications [14], or high-level CPS awareness [15], without systematically addressing sensor integration, control hierarchy placement, deployment realism, and trustworthiness in sensor-driven control as a whole.

At a deeper level, the relevance of LLMs in this area stems from a systems-integration challenge. Modern sensor-driven systems must synthesize heterogeneous sensor observations, human instructions, engineering documentation, domain knowledge, and control objectives within unified operational workflows. Classical control and estimation techniques remain essential for stability, robustness, and real-time execution, but they do not natively support open-ended language interaction, knowledge synthesis, or operator-facing explanation. LLMs offer a potentially valuable complementary layer for these functions. Their practical value, however, depends critically on how well they are grounded in sensor and system states, how they are integrated with existing control architectures, and how their outputs are constrained against nondeterminism and hallucination. These considerations motivate a focused review of LLMs in sensor-driven control systems.

### 1.2. Why This Review Is Needed

Research on LLMs in operational and cyber–physical settings is expanding rapidly, but it remains highly fragmented. Relevant work now appears across robotics [16], autonomous systems [17], industrial automation [10], digital twins [18], human–machine interaction [19], fault diagnosis [20], energy management [21,22], and broader cyber–physical systems research [23]. At the same time, closely related ideas are often developed under different labels and assumptions. Some studies present LLMs as planning agents [24], others as multimodal reasoning modules [25], engineering assistants [26], diagnostic interfaces [27], or supervisory decision-support tools [28]. This diversity reflects the broad appeal of LLMs, but it also makes it difficult to determine how they are actually being positioned within sensor-driven systems and which integration patterns are becoming most credible.

Existing reviews often capture only part of this broader landscape. Many focus primarily on robotics agents, embodied AI, or general-purpose LLM applications, with particular emphasis on instruction following, task planning, and interactive autonomy. Others examine LLMs from the perspective of software agents, foundation models, or multimodal intelligence without focusing on systems in which sensing, estimation, supervision, control, and actuation are operationally coupled. As a result, several issues central to sensor-driven control remain underexamined: how heterogeneous sensor information is represented for language-based reasoning, how LLM outputs relate to different layers of the control hierarchy, how such systems should be evaluated beyond language quality alone, and how they can be deployed safely under timing, reliability, and physical-world constraints.

A review organized explicitly around sensor-driven control is therefore needed to consolidate a dispersed literature, clarify the most credible roles of LLMs in such settings, and identify the architectural principles most likely to support useful and trustworthy adoption. Rather than treating LLMs simply as generic intelligent agents, this perspective emphasizes their interaction with sensing pipelines, state representations, planning and supervisory layers, conventional controllers, and human operators. It also highlights the need to distinguish among different forms of integration, ranging from offline engineering support and interpretive interfaces to supervisory decision support and closer-to-runtime participation. Equally important, it foregrounds evaluation and deployment criteria that are specific to operational systems, including grounding, robustness, safety, latency, explainability, and trustworthiness.

Unlike prior reviews that primarily focus on robotics, agentic systems, or specific application sectors, this review adopts the common systems perspective of sensor-driven control. It organizes the literature according to where and how LLMs are positioned within sensing–decision–control chains, with particular attention to architectural placement, methodological realization, application transferability, and operational trustworthiness. This framing also highlights a maturity gradient across LLM roles: functions that remain farther from direct actuation, such as interpretation and engineering support, are closer to practical deployment, whereas roles involving planning, supervision, monitoring, or runtime control participation require stronger grounding, verification, latency guarantees, and safety safeguards. Table 1 positions this review relative to prior surveys by comparing their quantitative scope and the core dimensions emphasized in this paper, namely sensor-driven coverage, role-oriented LLM analysis, architectural integration, methodological realization, cross-domain breadth, and evaluation/trust considerations.

### 1.3. Review Design and Analytical Scope

This paper adopts a structured review methodology that combines systematic literature identification with comparative qualitative synthesis. The search was conducted across Scopus, Web of Science, IEEE Xplore, ACM Digital Library, ScienceDirect, Google Scholar, and arXiv to capture relevant work on LLMs in robotics, cyber–physical systems, industrial automation, digital twins, autonomous systems, smart infrastructure, energy systems, and related sensor-driven domains.

The search focused on publications from the recent LLM era, particularly studies published from 2023 onward. Search terms combined LLM-related keywords, such as “large language model”, “LLM”, and “foundation model”, with system-level terms including “sensor-driven system”, “cyber-physical system”, “control system”, “robotics”, “industrial automation”, “digital twin”, “fault diagnosis”, “monitoring”, “planning”, “supervisory control”, and “human–AI interaction”.

The initial search identified 259 records. The retrieved records were screened through duplicate removal, title and abstract screening, and full-text eligibility assessment. Studies were retained when they presented, reviewed, or critically discussed the integration of LLMs with sensing, monitoring, estimation, supervision, planning, diagnosis, control, actuation, digital twins, robotic systems, industrial systems, or safety-relevant cyber–physical applications. Studies focused only on generic natural language processing, general chatbot use, perception-only pipelines without operational relevance, or internal LLM development without system-level integration were excluded. Following screening, 118 publications were retained and cited in the final qualitative synthesis.

Because the literature is heterogeneous in terminology, architecture, methodology, evaluation practice, and application domain, this review is conducted as a structured and conceptually guided synthesis rather than as a strict PRISMA-style systematic review or quantitative meta-analysis. The selected studies are analyzed through six dimensions: LLM role, architectural placement, sensor-to-semantics representation, integration pattern, application domain, and evaluation or trustworthiness considerations.

Table 2 summarizes the complete reference set used in the manuscript by publication year, application domain, and type of contribution. This includes the LLM-focused studies retained for qualitative synthesis as well as supporting references used for background, benchmarks, evaluation, and foundational sensor/control context.

### 1.4. Main Contributions of the Review

This review makes five main contributions. First, it introduces a role-based and maturity-aware taxonomy of LLM functions in sensor-driven control systems. The taxonomy distinguishes interpretive interfaces, supervisory decision support, planning and coordination, monitoring and diagnostics, engineering workflow support, and runtime control participation according to autonomy level, grounding demand, proximity to actuation, and deployment risk. Second, it synthesizes the principal architectural and methodological patterns through which LLMs are connected to sensing pipelines, state estimation, digital twins, external tools, human operators, and conventional control layers. Third, it compares representative application domains to identify cross-domain commonalities and domain-specific constraints in robotics, industrial automation, smart infrastructure, energy systems, healthcare, and other sensor-rich operational environments. Fourth, it develops an evaluation perspective tailored to operational systems, emphasizing grounding, robustness, latency, explainability, safety, security, and trustworthiness rather than language quality alone. Fifth, it identifies practical deployment pathways and future research directions for hybrid systems in which LLMs complement, rather than replace, classical control and estimation methods through structured representations, verification layers, fallback mechanisms, and human oversight.

**Figure 1:**
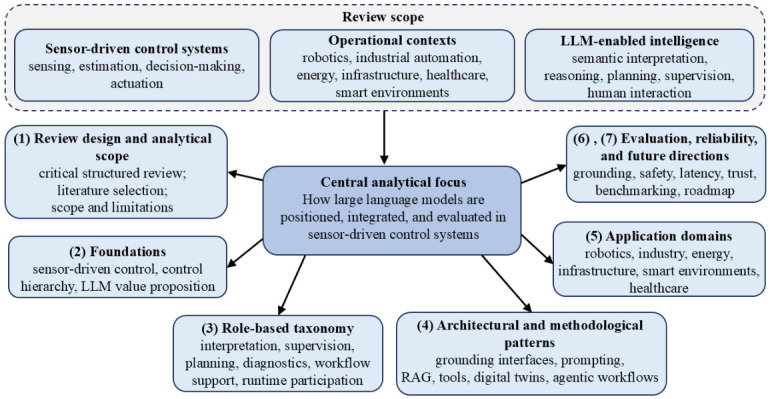
Review scope and organizing framework of this paper, showing the central analytical focus and the main dimensions used to synthesize the literature on LLMs in sensor-driven control systems.

The remainder of this paper is organized as follows. Section 2 introduces the conceptual foundation by tracing the progression from classical sensor-driven control systems to LLM-augmented operational architectures. Section 3 presents a taxonomy of LLM roles in sensor-driven control systems. Section 4 examines the architectural and methodological patterns through which LLMs are integrated into sensor-driven systems. Section 5 reviews representative application domains. Section 6 discusses evaluation frameworks, reliability gaps, and the need for more suitable benchmarking practices. Section 7 outlines promising future research directions. Finally, Section 8 concludes the paper. Figure 1 summarizes the review scope and organizing framework for LLMs in sensor-driven control systems.

## 2. Foundations: Sensor-Driven Control in the LLM Era

### 2.1. The Sensing–Estimation–Planning–Control–Actuation Cycle

Sensor-driven control systems operate on a closed-loop cycle that links sensing, estimation, planning, control, and physical execution. Sensors observe the plant, environment, or user; estimation modules transform raw measurements into usable representations of system state; planning and supervisory layers determine higher-level responses and coordination; and actuators apply those responses in the physical world. This cycle enables continuous adaptation to disturbances, uncertainty, and changing task demands.

In modern applications, sensing is often multimodal, combining structured telemetry with richer sources such as vision, LiDAR, audio, logs, alarms, and event traces. Because such inputs are frequently noisy, incomplete, or distributed across subsystems, they must be processed into state estimates, fault indicators, anomaly alerts, maps, or other task-relevant abstractions before action can be taken. This fundamental cycle underpins robotics, industrial automation, cyber–physical systems, smart grids, intelligent transportation, smart buildings, and infrastructure monitoring. Across these domains, reliable action depends on the timely and meaningful interpretation of measured system behavior.

**Figure 2:**
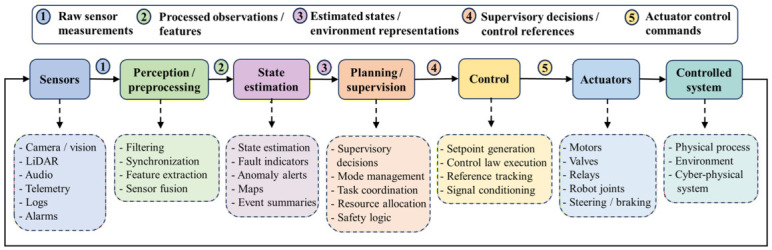
Closed-loop sensing–estimation–planning–control–actuation cycle in sensor-driven control systems.

Figure 2 illustrates this closed-loop sensing–estimation–planning–control–actuation structure. Raw multimodal sensor data are processed through perception and preprocessing modules, transformed by state estimation into higher-level abstractions, and then used by planning and supervisory layers to generate decisions and coordination strategies. The control layer converts these into executable commands, which are applied through actuators to the controlled process or environment, whose response closes the feedback loop.

### 2.2. Control Hierarchies

Sensor-driven systems are rarely organized as a single control loop. Instead, they are typically structured as hierarchies with different temporal and semantic layers. At the lowest level, feedback regulation maintains stability, tracks references, and rejects disturbances through fast controllers such as proportional–integral–derivative (PID) and model predictive control (MPC) [31,32]. Above this layer, supervisory mechanisms manage modes, constraints, alarms, fallback actions, and subsystem coordination. Planning layers operate over longer horizons, handling task decomposition, sequencing, subgoal generation, and adaptation to changing contexts. Human-facing layers support natural-language interaction, explanation, querying, and decision support.

This hierarchical organization is essential for understanding where LLMs may fit. Low-level regulation requires deterministic timing, numerical reliability, and formal guarantees under physical constraints. By contrast, supervisory, planning, and human-facing layers depend more heavily on semantic interpretation, contextual reasoning, and flexible communication, making them much more compatible with LLM-based support [33,34].

### 2.3. What LLMs Add

LLMs contribute capabilities that are largely absent from conventional sensing, estimation, and control pipelines. Classical control methods excel at numerical regulation, prediction, and constraint-aware execution, but they do not naturally interpret open-ended language, synthesize dispersed textual knowledge, explain recommendations in human-readable form, or flexibly connect heterogeneous operational information to human goals. LLMs add value primarily at this semantic and contextual layer [35,36].

In sensor-driven systems, they can help interpret telemetry, alarms, logs, reports, and multimodal summaries; connect observations with procedural knowledge, prior events, and operator intent; generate explanations for system behavior or recommendations; support higher-level planning through task decomposition and sequencing; and coordinate access to external tools such as simulators, databases, digital twins, planners, and verification modules [35,36]. Their main value lies in augmenting operational pipelines with semantic interpretation, contextual reasoning, explanation, planning support, and tool-mediated coordination.

### 2.4. Guiding Perspective

A guiding perspective of this review is that LLMs are most credible in sensor-driven control systems when they are used as supervisory, interpretive, diagnostic, and human-facing intelligence layers rather than as direct replacements for classical control [36,37]. Reliable sensing, robust estimation, deterministic feedback, and timely actuation remain essential for maintaining stability, satisfying constraints, and ensuring safe physical operation. Classical controllers and estimation methods remain indispensable because they are specifically designed for these requirements.

LLMs are most effective where semantic interpretation, knowledge integration, flexible communication, and higher-level reasoning are needed. In the most credible architectures emerging in the literature, they are layered on top of sensing, estimation, and control foundations to summarize system state, assist diagnosis, support supervisory decisions, translate operator goals into machine-relevant objectives, and coordinate planning or tool use. By contrast, direct use of LLMs in low-level control remains far less mature and raises serious concerns regarding latency, nondeterminism, weak physical grounding, hallucination, and the absence of formal guarantees [37]. The most promising path forward is therefore not end-to-end language-driven control, but hybrid architectures in which LLMs complement rather than displace the foundations of control engineering.

Taken together, these foundations suggest that the key question is not whether LLMs can be added to sensor-driven systems, but which roles they can perform credibly within existing control architectures.

## 3. A Taxonomy of LLM Roles

This section presents a taxonomy of LLM roles in sensor-driven systems. Rather than organizing roles by model architecture or application domain, we classify them according to their functional position within the sensing–decision–control chain, specifically in terms of how their outputs relate to sensing, supervisory reasoning, decision-making, and execution.

The taxonomy distinguishes roles primarily by their function and operational consequences. Some roles emphasize interpretation and situational awareness; others support supervisory decision-making, planning and coordination, diagnostics, engineering workflows, or runtime control participation [10,12,35]. Although these roles may overlap in practice, they differ substantially in their implications for system design, grounding requirements, and deployment risk.

To highlight these differences, each role is evaluated along three cross-cutting dimensions:

- Proximity to actuation—how directly the LLM’s outputs influence physical system behavior;
- Grounding demand—the degree to which outputs must remain tightly linked to sensor data and system state;
- Deployment risk—the potential consequences of errors, hallucinations, latency, or misinterpretation [37].

This functional taxonomy provides a consistent framework for comparing studies that often use different terminology and clarifies which roles are currently the most mature and defensible in sensor-driven systems. The roles are presented from lower-risk interpretive and supervisory functions to higher-risk roles that approach direct runtime control.

To make the taxonomy more concrete, the following subsections connect each role to representative case-oriented examples from the reviewed literature. These examples include autonomous-driving scene interpretation, operator-facing supervisory assistance, scene-graph-grounded robot planning, telemetry- and log-based diagnostics, programmable logic controller (PLC)/control-code engineering support, and LLM-assisted control adaptation [36,38,39,40,41,42,43,44,45,46,47]. Because many current studies remain at the prototype, simulation, or experimental-validation stage, they are used here as operationally relevant examples rather than as evidence that all roles are fully mature or deployed in practice. Across the subsections, the examples are discussed in terms of practical value, proximity to actuation, deployment risk, and required safeguards.

### 3.1. Interpretation and Situational Awareness

One of the most credible and practically useful roles of LLMs in sensor-driven systems is interpretation and situational awareness. In this role, they act as an interpretive layer that transforms heterogeneous and often fragmented operational data into a coherent understanding of the current system state, ongoing processes, and emerging issues [10,35,38].

A core function is the contextualization of telemetry and state information. Sensor-driven systems generate continuous streams of measurements related to motion, energy consumption, process variables, and component health. LLMs can organize these data into higher-level summaries, identify meaningful patterns across multiple signals, and connect numerical observations to the operational context, thereby bridging raw data and human- or supervisor-level understanding [38,39].

LLMs also support the interpretation of alarms, logs, and event traces. Operational environments frequently produce large volumes of warnings, fault codes, and asynchronous events that are difficult to interpret in isolation. LLMs can synthesize these sources into coherent narratives, highlight probable relationships among events, and help distinguish between isolated anomalies and broader systemic issues [10,48].

Another valuable capability is the integration of multimodal observations. In many applications, situational awareness requires combining numerical telemetry with visual data, inspection reports, and operator notes. When coupled with perception models, LLMs can fuse these heterogeneous sources into a unified operational picture. This is particularly useful in robotics, industrial monitoring, infrastructure inspection, and autonomous systems [38,39,49].

More broadly, LLMs contribute to high-level situational reasoning by generating concise state summaries, explaining noteworthy deviations, and framing system status in relation to operational goals and recent history. Their strength lies not in replacing dedicated estimators or perception modules, but in providing semantic integration and context-aware interpretation of their outputs [35,38].

The effectiveness of this role depends heavily on grounding. Unconstrained interpretations risk hallucination or oversimplification. Therefore, the most reliable implementations rely on structured state representations, retrieval-augmented generation (RAG), perception pipelines, or human verification to ensure interpretations remain faithful to actual system conditions [10,38].

Overall, interpretation and situational awareness constitute one of the strongest near-term applications of LLMs in sensor-driven systems. The role is operationally valuable across domains, relatively low-risk compared to runtime control, and serves as a natural bridge between raw sensor data and higher-level supervisory decision-making. As such, it forms a key component in hybrid architectures where conventional sensing and control mechanisms remain responsible for execution while LLMs enhance transparency and understanding [10,35,38].

**Figure 3:**
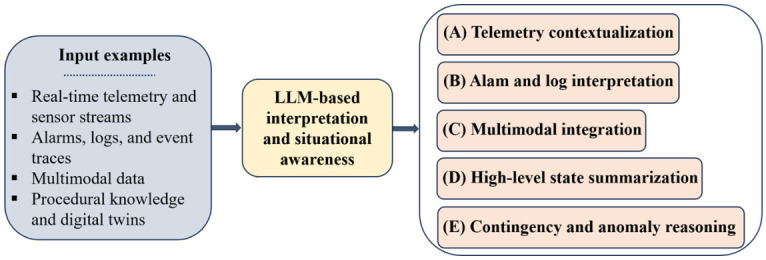
LLM-based interpretation and situational awareness in sensor-driven systems. The LLM acts as an interpretive layer that transforms heterogeneous sensor data, alarms, logs, and multimodal observations into coherent, context-aware understanding.

Figure 3 illustrates the role of LLMs in interpretation and situational awareness.

### 3.2. Supervisory Decision-Support

A central and highly credible role of LLMs in sensor-driven systems is supervisory decision support. In this role, LLMs operate at an intermediate layer above direct control execution. They assist operators and automated supervisory systems by interpreting complex situations, prioritizing responses, and supporting higher-level operational decisions without issuing low-level actuator commands [12,35].

One key function is mode selection. Many systems operate under multiple supervisory modes, such as normal, degraded, fault-recovery, safe, or manual override. Selecting the appropriate mode requires integrating sensor observations, system context, procedural rules, and safety constraints. LLMs can support this process by reasoning across heterogeneous information sources and recommending the most suitable mode under current conditions [39,40].

A second important function is alarm prioritization. Operational environments often generate large volumes of alarms and warnings, many of which are redundant or low-priority. LLMs can contextualize alarms against overall system state, historical patterns, and operational objectives, thereby helping operators and supervisory logic focus attention on the most critical signals [10,41].

LLMs also contribute to exception handling. When systems encounter situations outside routine assumptions, such as sensor failures, communication disruptions, or conflicting subsystem demands, LLMs can organize available evidence, identify plausible response options, and support flexible decision-making when predefined workflows are insufficient [39,40].

Another valuable capability is contingency reasoning. LLMs can anticipate possible future developments, evaluate alternative scenarios, and suggest fallback strategies or recovery options under uncertainty and partial observability. This forward-looking support is particularly useful for supervisory decision-making in dynamic environments [50,51].

The primary strength of LLMs in supervisory decision-support lies in their ability to integrate heterogeneous sensor-derived evidence with procedural knowledge, operational context, and human-centered reasoning. They translate raw observations, alarms, and system events into meaningful supervisory judgments. Importantly, execution remains delegated to conventional controllers, safety systems, or human operators, thereby maintaining a clear separation from direct control [39,40].

Consequently, supervisory decision-support represents one of the most practical and defensible applications of LLMs in sensor-driven systems. It occupies a productive middle ground between purely assistive roles and high-risk runtime participation, enabling semantic interpretation and structured reasoning while preserving safety and reliability through established control mechanisms [12,35,51].

**Figure 4:**
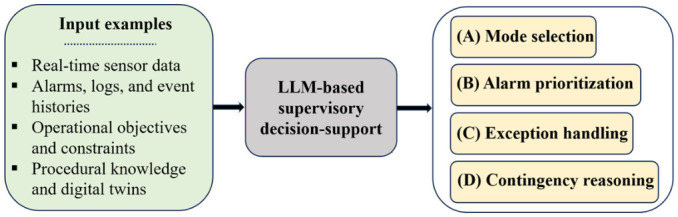
LLM-based supervisory decision-support in sensor-driven systems. The LLM operates at an intermediate supervisory layer, assisting with mode selection, alarm prioritization, exception handling, and contingency reasoning while leaving low-level execution to conventional controllers or human operators.

Figure 4 illustrates the role of LLMs in supervisory decision support.

### 3.3. Task Planning and Coordination

LLMs serve as supervisory layers for task planning and coordination in sensor-driven systems. Operating above low-level control loops, they integrate high-level user goals, real-time sensor data, structured world models, and operational constraints to generate structured sequences of subtasks and coordinated behaviors across modules, agents, or robot teams [30,35,52,53,54].

A core function is goal decomposition. Industrial and robotic tasks are often too abstract for direct execution. LLMs break them into actionable subgoals for perception, planning, or control modules. This process becomes significantly more reliable when grounded in scene graphs, symbolic models, or digital twins [42,52,55,56].

LLMs also support task sequencing by organizing subtasks into coherent workflows. In industrial and cyber–physical systems, this includes coordinating monitoring, diagnosis, alarm handling, and maintenance procedures while respecting temporal dependencies and contextual constraints. Owing to their strength in symbolic and linguistic reasoning, LLMs effectively handle procedural knowledge and conditional instructions, thereby complementing numerical optimization methods [52,57,58,59].

Replanning and adaptation is another key capability. Sensor-driven systems frequently encounter changing conditions due to new observations, faults, operator interventions, or resource constraints. LLMs can revise action sequences, propose alternative subgoals, or generate contingency plans by integrating sensor data with procedural and human-intent knowledge. Recent approaches leverage iterative feedback from digital twins and runtime monitors to enhance robustness [55,60].

In multi-agent and multi-module architectures, LLMs serve as coordination layers. They mediate communication, allocate subtasks, maintain coherence, and support collaborative execution across perception modules, digital twins, robot teams, and human supervisors [61,62,63].

The supporting modules, strengths, and limitations of these functions are summarized in Table 3. Despite these strengths, LLMs do not inherently guarantee optimality, feasibility, temporal correctness, or safety. Their value lies primarily in high-level abstraction, semantic mediation, and contextual adaptation. Therefore, the most robust deployments integrate LLMs within hybrid neuro-symbolic architectures, where their outputs are grounded by explicit world models or digital twins and constrained by symbolic planners, simulators, verification layers, or human-in-the-loop oversight [42,52,55].

**Figure 5:**
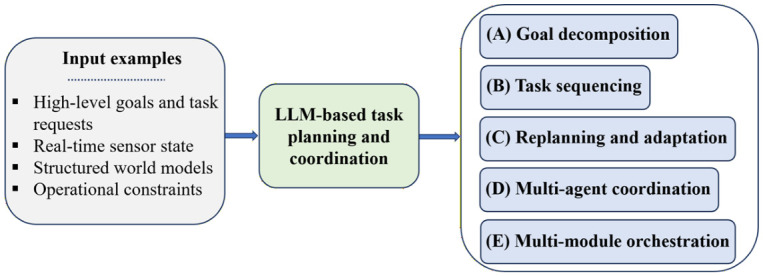
Overview of LLM-based task planning and coordination in sensor-driven systems. The LLM acts as a supervisory layer that processes user goals, sensor data, world models, and operational constraints to support goal decomposition, sequencing, adaptation, coordination, and orchestration, ultimately producing an executable operational plan.

Figure 5 shows an overview of LLM-based task planning and coordination in sensor-driven systems.

### 3.4. Monitoring and Diagnostics

LLMs serve as an interpretive supervisory layer in sensor-driven systems for monitoring and diagnostics. Rather than performing low-level detection, they integrate heterogeneous data sources such as telemetry streams, alarms, logs, maintenance records, and operator notes to explain abnormal behavior, support root-cause analysis, and assist maintenance decision-making [10]. Recent reviews of large-model-based machine monitoring and fault diagnostics further categorize emerging approaches into in-context learning, fine-tuning, retrieval-augmented generation, multimodal learning, and time-series-based methods, highlighting their potential for adaptive and interpretable PHM systems while also emphasizing challenges related to industrial data availability and edge deployment [64].

A primary contribution is root-cause support. LLMs can synthesize evidence from alarm sequences, sensor trends, historical records, and procedural knowledge to generate plausible diagnostic hypotheses. They complement conventional fault-detection methods by organizing multi-source evidence and comparing alternative explanations [43,65,66,67].

LLMs are also effective at anomaly contextualization and fault explanation. After detection, they assess the significance of an anomaly, relate it to prior events or known failure modes, and translate technical fault signals into human-understandable narratives. This helps operators distinguish between transient disturbances, sensor issues, and emerging faults [10,65].

Another relevant capability is predictive maintenance support. LLMs integrate condition-monitoring data with inspection findings, failure histories, spare-part availability, scheduling constraints, and risk assessments to prioritize interventions and generate actionable recommendations [44,68,69].

Finally, LLMs support diagnostic workflow coordination by orchestrating interactions among monitoring tools, digital twins, knowledge bases, and human experts [58,63].

The main diagnostic functions, their supporting inputs/modules, strengths, and limitations are summarized in Table 4. Despite these strengths, LLM-based diagnostics require rigorous grounding. Fluent but unverified explanations risk incorrect conclusions or oversimplification. The most reliable implementations therefore embed LLMs in hybrid workflows, where their reasoning is constrained by sensor-derived evidence, engineering models, and human verification [10,44,65,70,71].

**Figure 6:**
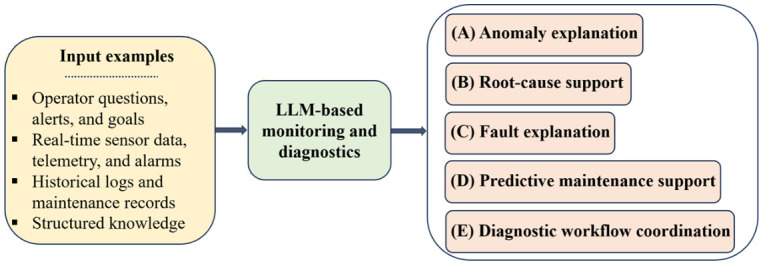
Overview of LLM-based monitoring and diagnostics in sensor-driven systems. The LLM acts as an interpretive layer that processes heterogeneous inputs to support anomaly contextualization, root-cause analysis, fault explanation, predictive maintenance, and diagnostic workflow coordination.

Figure 6 illustrates the overall workflow of LLM-based monitoring and diagnostics.

### 3.5. Engineering Workflow Support

LLMs provide valuable assistance in the engineering workflows that surround sensor-driven systems. Rather than acting as runtime controllers, they support offline and semi-offline tasks such as code generation, commissioning, documentation, configuration, debugging, and troubleshooting [36,45].

A major contribution is code generation and revision. LLMs can generate control logic, PLC programs, interface scripts, and configuration files, while also explaining existing code and suggesting improvements. Studies demonstrate notable productivity gains in industrial automation and robotics, although formal verification remains essential [45,72,73].

LLMs also support system commissioning and setup. These phases involve signal mapping, parameter configuration, device integration, and alignment of hardware/software under strict time and safety constraints. LLM-based assistants can interpret documentation, generate configuration guidance, and facilitate complex integration procedures [36,74].

Another key capability is documentation and knowledge support. LLMs can summarize technical manuals, extract relevant procedures, answer engineering queries, and link documentation with digital twins, thereby improving access to dispersed engineering knowledge [44,75].

LLMs also offer effective configuration and troubleshooting support. They help diagnose integration errors, misconfigured interfaces, and software faults by interpreting logs, suggesting debugging steps, and organizing diagnostic information [10,74].

Table 5 summarizes the main forms of engineering workflow support provided by LLMs in sensor-driven systems.

One of the primary advantages of this role is its relatively low-risk profile. Since LLM outputs can be inspected, tested, and verified by engineers before deployment, engineering workflow support allows safe use of LLM capabilities while maintaining human oversight and formal validation. This makes it one of the most practical near-term applications of LLMs in sensor-driven systems [36,45].

**Figure 7:**
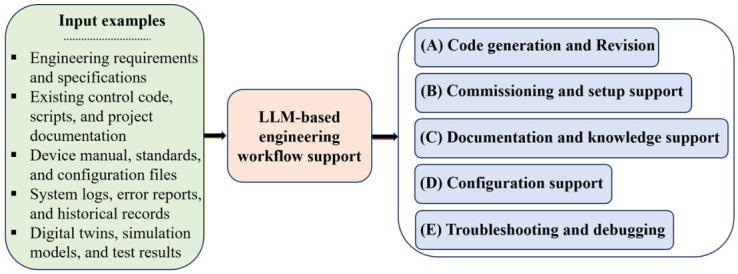
Overview of LLM-based engineering workflow support in sensor-driven systems. The LLM assists with code generation, commissioning, documentation, configuration, and troubleshooting by transforming engineering inputs and operational records into validated workflow outputs under human oversight and formal checking.

Figure 7 illustrates the conceptual workflow of LLM-based engineering support in sensor-driven systems, showing how design inputs, technical documentation, and operational records are transformed into code, commissioning guidance, configuration recommendations, and troubleshooting outputs under validation and human oversight.

### 3.6. Runtime Control Participation

The most control-proximal and highest-risk role of LLMs in sensor-driven systems is runtime control participation, where model outputs influence operational decisions during execution. Examples include controller-parameter adjustment, set-point recommendation, constrained action selection, execution-time replanning, and control-logic assistance for robotic or industrial systems [37,45,46,76].

One prominent form is parameter tuning. LLMs can interpret performance indicators, relate them to control objectives, and propose adjustments to controller gains, thresholds, reference values, or supervisory parameters. In this setting, the LLM acts as a higher-level adaptive layer rather than replacing the underlying feedback controller [33,46].

A second form is constrained action recommendation. Instead of issuing unconstrained low-level commands, the LLM proposes actions within a bounded operational envelope enforced by safety filters, optimization layers, rule-based constraints, or supervisory logic. This is relevant in safety-critical applications such as autonomous marine vessels, multi-UAV systems, and industrial robotics, where semantic reasoning must remain coupled with physical and operational constraints [37,47,76].

Runtime involvement may also include execution-time replanning, dynamic task prioritization, and trajectory-level reasoning triggered by sensed anomalies or environmental changes. For example, recent robotic manipulation studies investigate whether LLMs can generate dense end-effector trajectories from language instructions and visual perception inputs. These studies indicate emerging capability in trajectory-level reasoning, but they also highlight the need for feasibility checking, failure detection, controller-level validation, replanning, and safety filtering before physical execution [77].

Unlike offline planning or diagnosis, runtime outputs may affect near-term physical behavior with limited time for correction. This makes latency, output variability, weak physical grounding, infeasible actions, and lack of formal stability guarantees particularly critical. Therefore, such systems require stronger assurance mechanisms than interpretive, diagnostic, or offline engineering-support applications.

Table 6 synthesizes the main forms of runtime control participation identified in the reviewed literature, together with their advantages, risks, and required safeguards.

Despite growing research interest, this remains the least mature form of LLM integration in sensor-driven systems. Close proximity to the control loop can amplify weaknesses such as inference delay, hallucination, nondeterminism, and limited grounding, allowing them to propagate from symbolic reasoning to unsafe operational behavior.

Credible implementations therefore require constrained hybrid architectures in which LLM outputs are treated as proposals rather than executable commands. Before execution, they should be checked by constraint validators, verification layers, optimization modules, runtime monitors, or validated controllers. If an output is delayed, infeasible, uncertain, inconsistent with the measured state, or outside predefined limits, the system should revert to a verified baseline controller, safe operating mode, safe shutdown procedure, or human-supervised state [33,34,37,46,47,78].

Human approval remains important when model outputs may affect safety-critical operations, maintenance actions, clinical decisions, or irreversible system changes. Depending on the application, this may involve operator confirmation, engineering review, permission levels, or supervisory authorization. Runtime decisions should also be logged to support traceability, post-event analysis, accountability, and reproducibility.

**Figure 8:**
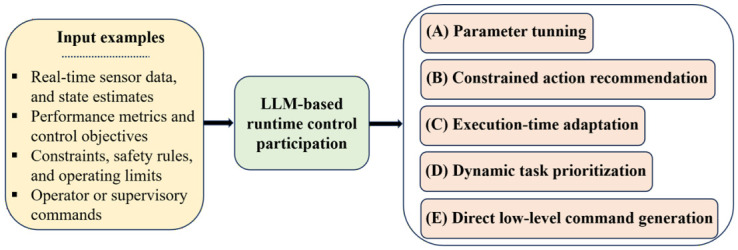
Runtime control participation of LLMs in sensor-driven systems. The LLM may influence operational decisions through parameter tuning, constrained action recommendation, execution-time replanning, or other execution-adjacent interventions. Such participation requires bounded authority, safety filters, fallback mechanisms, verification layers, and human or supervisory approval before affecting physical operation.

Overall, LLM-based runtime participation should be regarded as a frontier research direction rather than a default integration strategy. Its potential for semantic and adaptive behavior is significant, but current evidence supports its use only when the model’s authority is bounded, auditable, interpretable, and subordinate to reliable sensing, estimation, verification, and control mechanisms [37,47]. Figure 8 summarizes this role and its main safety requirements.

### 3.7. Comparative Synthesis

Taken together, the roles examined in this taxonomy reveal a clear maturity gradient in LLM integration for sensor-driven systems. At the lower-risk end lie interpretation and situational awareness, supervisory decision-support, and engineering workflow assistance. These roles remain relatively distant from direct actuation, allow substantial human oversight, and permit outputs to be inspected, revised, or rejected before influencing physical behavior. They therefore represent the most credible and immediately deployable applications of LLMs today.

Interpretation and situational awareness are among the most mature, as they transform fragmented sensor data, alarms, logs, and multimodal inputs into coherent operational understanding without closing the control loop. Supervisory decision support is similarly robust, providing valuable assistance in mode selection, alarm prioritization, exception handling, and contingency reasoning while keeping execution with conventional controllers or human operators. Engineering workflow support benefits from operating largely outside the runtime loop, where validation is straightforward.

Planning and coordination roles occupy an intermediate position. They deliver significant value by bridging high-level goals with structured plans and multi-agent activities, but they increase grounding and verification demands, since planning errors can propagate downstream. Their success therefore depends on strong hybrid architectures incorporating symbolic planners, simulators, and verification mechanisms.

At the highest-risk end lies runtime control participation, including parameter tuning, constrained action recommendation, and other execution-adjacent interventions. These roles come closest to direct actuation and therefore impose the strictest requirements for safety assurance, determinism, and physical grounding. Current evidence suggests they remain the least mature and most difficult to justify for real-world deployment, except in tightly constrained hybrid setups.

This risk gradient highlights a central insight: LLM integration in sensor-driven systems is not a binary choice. Different roles entail fundamentally different trade-offs in risk, grounding needs, and validation effort. The most defensible near-term pathway is the progressive adoption of lower-risk interpretive, supervisory, and engineering roles that enhance transparency and human effectiveness while preserving established control foundations.

In summary, the taxonomy advocates a layered, risk-aware approach. Roles farther from actuation are currently more mature, trustworthy, and easier to integrate. Roles closer to runtime control offer greater adaptive potential but remain frontier research directions that demand substantially stronger safeguards before practical deployment.

**Figure 9:**
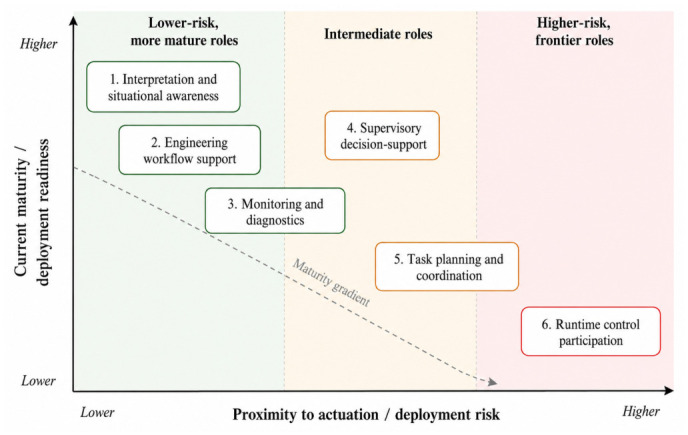
Maturity gradient of LLM roles in sensor-driven systems, from lower-risk interpretive and workflow roles to higher-risk runtime control participation.

Figure 9 illustrates the maturity landscape of LLM roles in sensor-driven systems as a function of proximity to actuation and deployment risk.

## 4. Architectural and Methodological Integration Patterns

This section examines the main architectural and methodological patterns through which LLMs are integrated into sensor-driven control systems. It considers where LLMs are positioned within control architectures, how LLMs interoperate with legacy automation and control infrastructure such as PLCs and supervisory control and data acquisition (SCADA) systems, how sensor and operational data are transformed into LLM-usable contexts, how LLM capabilities are implemented in practice, how external tools and hybrid modules are coupled with language-based reasoning, and how feedback and safety mechanisms constrain model influence for trustworthy deployment.

### 4.1. Architectural Placement

A central question in LLM integration is not only what role the model performs, but where it is positioned within the overall system architecture. Architectural placement determines what information the LLM can access, what outputs it can generate, how directly those outputs influence system behavior, and which validation or safety mechanisms are required [35,36].

LLM placement spans a broad spectrum. At one end, models operate far from the runtime loop, for example as human-facing assistants, documentation tools, or engineering support systems. These positions are relatively low-risk because outputs can be reviewed, revised, or rejected before affecting physical execution. At the other end, LLMs are placed closer to planning, supervision, or runtime decision-making, where their outputs may more directly influence operational behavior. Such placements offer greater adaptive potential but require stronger grounding, constraints, and safety assurance [37,38].

Key architectural interfaces include:

- Sensing layer: LLMs are rarely applied directly to raw sensor streams. Instead, they typically operate downstream of preprocessing, fusion, or abstraction layers, consuming structured representations such as state summaries, scene graphs, alarm groups, or event descriptions [39].
- State estimation and representation: Placement after estimation modules enables the LLM to reason over coherent, higher-level system states, improving interpretability while reducing sensitivity to noisy or incomplete observations [39].
- Planning and supervisory layers: This is one of the most common and credible placements. Here, LLMs support goal decomposition, mode selection, alarm prioritization, exception handling, and contingency reasoning, while low-level execution remains delegated to conventional controllers [36,47].
- Control loops: Most credible architectures keep LLMs outside fast inner control loops. Direct insertion into timing-critical loops is generally avoided because these layers require determinism, low latency, numerical reliability, and formal guarantees [37].
- Digital twins and external modules: Positioning the LLM alongside simulators, planners, optimizers, databases, and verification tools enables it to function as an orchestration layer that queries, interprets, and integrates outputs from specialized components [36].
- Human operators and safety layers: In supervisory systems, the LLM often mediates between technical subsystems and human users. Robust designs place safety filters, validation wrappers, fallback policies, and human approval mechanisms between LLM outputs and physical execution [37].

Overall, architectural placement is one of the most important design decisions in LLM-enabled sensor-driven systems. Positions farther from direct actuation generally provide lower risk and higher deployment feasibility, whereas placements closer to planning and control increase adaptive capability but also raise grounding and validation demands. Effective integration therefore requires careful co-design of the LLM layer with sensing, estimation, control, supervision, and safety mechanisms.

### 4.2. Interoperability with Legacy Automation and Control Infrastructure

A practical barrier to deploying LLMs in sensor-driven control systems is interoperability with existing automation infrastructure, including PLCs, SCADA systems, distributed control systems (DCSs), building management systems (BMSs), human–machine interfaces (HMIs), industrial historians, and field-level communication protocols. These infrastructures are typically designed around deterministic execution, validated control logic, strict timing requirements, role-based access, and auditable operator interaction. By contrast, LLMs are probabilistic reasoning modules whose outputs may vary with prompts, retrieved context, model versions, and interaction history. This mismatch creates integration challenges related to latency, reliability, accountability, cybersecurity, and actuation safety [36,79].

A defensible integration strategy is to connect the LLM through controlled middleware, APIs, or supervisory interfaces rather than allowing direct access to low-level control logic. In low-risk configurations, the LLM may operate with read-only access to alarms, logs, historian data, maintenance records, manuals, or digital-twin outputs. If LLM-generated outputs are allowed to influence operational actions, such as setpoint changes, maintenance actions, or schedule updates, they should pass through schema validation, engineering rules, permission controls, safety constraints, human approval, and audit logging before being applied. In this architecture, the LLM supports interpretation and supervisory reasoning, while established automation layers retain responsibility for real-time control, interlocks, alarms, emergency shutdowns, and safety-critical functions [36,80].

Another key challenge is semantic grounding. Legacy systems often encode process knowledge through tag names, alarm codes, ladder logic, function blocks, piping and instrumentation diagrams, control narratives, and vendor-specific data structures. These representations are not automatically meaningful to an LLM. Effective integration therefore requires a grounding layer that maps sensor tags, actuator states, operating modes, alarms, and procedures into structured and traceable representations. Such grounding can be supported by asset models, knowledge graphs, ontologies, digital twins, retrieval-augmented access to manuals, or standardized industrial information models. This reduces the risk that LLM outputs remain linguistically plausible but weakly connected to the actual plant state [36,79].

Overall, interoperability with PLC, SCADA, DCS, BMS, HMI, and historian systems reinforces the need for hybrid architectures. Near-term deployment is most credible at supervisory timescales, where LLMs can assist with alarm explanation, fault interpretation, operator guidance, maintenance planning, documentation, code review, and what-if analysis. Fast inner-loop control, protection functions, and emergency responses should remain within deterministic controllers and verified automation logic.

### 4.3. Sensor-to-Semantics Pipeline

A fundamental challenge in integrating LLMs into sensor-driven systems is that raw sensor data are rarely suitable for direct language-model input. Sensor-driven environments generate high-dimensional, noisy, asynchronous, and heterogeneous streams, including time-series telemetry, alarms, logs, images, point clouds, and estimated state variables. Effective integration therefore depends on a sensor-to-semantics pipeline: a set of techniques that transform raw operational data into structured and context-rich representations suitable for LLM reasoning [36,38,39].

The pipeline typically begins with preprocessing and abstraction. Raw measurements are filtered, aggregated, and converted into compact, semantically meaningful forms such as state summaries, event descriptions, alarm groups, scene graphs, or fault indicators. This stage reduces data volume while preserving operational relevance through feature extraction, temporal aggregation, and multimodal fusion [38].

The next stage is state representation. Abstracted data must be organized into formats that support language-based reasoning, such as textual state descriptions, structured tables, symbolic summaries, or hybrid prompts that combine numerical values with explanatory context. The quality of this representation strongly influences the coherence, relevance, and faithfulness of LLM outputs [39,49].

A further stage is context construction. Current observations are rarely sufficient on their own. The pipeline therefore augments real-time state information with historical context, procedural knowledge, mission objectives, digital-twin outputs, or operator instructions. This allows the LLM to interpret the current situation as part of an evolving operational process [10,45].

In multimodal systems, which are common in robotics, autonomous platforms, and industrial monitoring, the pipeline must align numerical telemetry with visual, textual, or descriptive data. This cross-modal integration is usually performed by dedicated perception and fusion modules upstream of the LLM, rather than requiring the LLM to process raw multimodal streams directly [38,39].

The sensor-to-semantics pipeline is therefore a core architectural component rather than a minor implementation detail. It determines how effectively raw sensing is translated into operational meaning, how well the LLM remains grounded in actual system conditions, and how safely its outputs can be used by supervisory or control layers. In practice, the success of LLM integration often depends as much on this representation pipeline as on the model itself [36].

### 4.4. Methodological Realization

**Figure 10:**
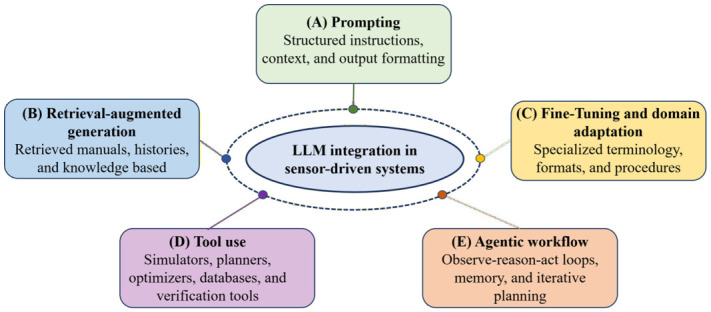
Methodological realization for LLM integration in sensor-driven systems.

Methodological realization determines how an LLM role is technically implemented in practice. Across the literature, LLM integration in sensor-driven systems commonly relies on five methodological patterns: prompting, retrieval-augmented generation (RAG), fine-tuning, tool use, and agentic workflows shown in Figure 10. These patterns differ in complexity, controllability, robustness, engineering effort, and deployment implications, but they share a common aim: connecting the LLM to operational data and system context rather than using it as an isolated language model [35,36,45].

The simplest and most widely used pattern is prompting. Structured instructions, contextual information, and output constraints guide the LLM to generate summaries, explanations, recommendations, or plans. Prompting is flexible and inexpensive to implement, but prompt-only systems are sensitive to input quality and can become brittle when context is incomplete, noisy, or poorly structured [35,45,81].

A more robust pattern is RAG. This approach augments the prompt with relevant external knowledge retrieved from manuals, procedures, maintenance histories, digital twins, or domain-specific databases. RAG is especially useful in sensor-driven systems, where effective reasoning often requires technical background knowledge that is not contained in raw sensor streams [10,36].

Fine-tuning and domain adaptation provide another implementation path. By adapting pretrained models to domain-specific terminology, procedures, or response formats, fine-tuning can improve consistency and alignment for recurring tasks. However, it increases engineering effort and raises concerns regarding data quality, maintenance, and generalization [36].

Tool use is becoming increasingly important. In this pattern, the LLM acts as an orchestrator that invokes external modules such as simulators, planners, optimizers, databases, or verification tools. Tool use allows the system to combine language-based reasoning with reliable, deterministic computation [10,36,82].

The most advanced pattern is agentic workflow design. Here, the LLM operates within an explicit observe–reason–act loop, often incorporating memory, iterative planning, self-correction, and multi-step tool coordination. Agentic systems provide high flexibility for complex sequential tasks, but they also increase latency, complexity, and the need for monitoring and failure recovery [38,45].

These patterns are rarely used in isolation. State-of-the-art systems typically combine structured prompting, retrieved knowledge, tool calling, and iterative refinement. Methodological choices should therefore be regarded as part of the system architecture, since they directly shape grounding quality, reliability, and deployment feasibility.

### 4.5. Tool-Augmented and Hybrid Integration

A recurring pattern in LLM-enabled sensor-driven systems is tool-augmented and hybrid integration. Rather than relying on the LLM as a standalone reasoning engine, these architectures couple it with specialized external modules that perform functions the LLM cannot reliably or efficiently handle alone. In such designs, the LLM contributes semantic interpretation, contextual reasoning, and high-level coordination, while external components provide structured computation, system modeling, simulation, optimization, and validation [10,36,45,82].

Common external modules include:

- Simulators and digital twins: These modules allow candidate actions, plans, or predictions to be evaluated in virtual environments before real-world execution. This improves grounding and reduces operational risk by linking language-based reasoning to plant- or mission-specific models [82].
- Planners and optimization engines: The LLM can formulate problems, decompose goals, and interpret constraints, while feasible plans or optimized solutions are generated by classical planners and solvers. This preserves the strengths of formal methods while using the LLM for semantic flexibility [45].
- Formal verification and runtime monitoring tools: In safety-critical applications, LLM outputs can be checked by rule engines, constraint validators, runtime monitors, or formal verifiers. These components can reject unsafe suggestions, request reformulation, or enforce operational boundaries [37].

In hybrid architectures, the LLM typically functions as an orchestration layer. It interprets the current situation, selects appropriate tools, invokes them, and integrates their outputs into coherent recommendations or supervisory decisions. This role allows the system to combine the flexibility of language-based reasoning with the reliability of specialized computational components.

Tool-augmented and hybrid integration is therefore one of the most credible pathways for deploying LLMs in sensor-driven systems. It avoids the unrealistic expectation that LLMs should replace established estimation, planning, or control components. Instead, it positions the LLM as a semantic and supervisory intermediary within a broader technical stack, where critical functions remain grounded in domain-specific tools with stronger guarantees of correctness, feasibility, and safety [36,82].

### 4.6. Closed-Loop Refinement and Self-Correction

Another important pattern is closed-loop refinement and self-correction. Rather than treating the LLM as a one-shot generator, these approaches iteratively assess its outputs against external feedback and prompt the model to revise its reasoning, recommendations, code, or plans. This is particularly valuable in sensor-driven environments, where operational validity depends on consistency with execution results, simulator behavior, safety constraints, and evolving system state [12,45,83].

One important source of feedback is execution feedback. When an LLM-generated action, plan, or code fragment is executed, resulting outcomes such as compiler errors, task failures, interface mismatches, or degraded performance can be returned to the model. This enables evidence-based correction rather than relying solely on the initial output [45,84].

A second source is simulator and digital-twin feedback. Proposed actions or plans can be tested in virtual environments before real-world deployment. Feedback from simulators or digital twins helps the LLM evaluate predicted consequences and refine its outputs in a lower-risk setting [12,83].

A third source is constraint violation and rule-based rejection. Outputs can be checked against logical, temporal, physical, or safety constraints. Invalid suggestions may then be rejected or returned to the LLM with structured error signals for reformulation [37,85].

A fourth mechanism is self-reflection and iterative revision. In these cases, the LLM is prompted to critique its own outputs, identify weaknesses, or generate improved versions. However, self-reflection is most useful when anchored by external evidence rather than performed in isolation [83].

Closed-loop refinement is important because many sensor-driven tasks cannot be solved reliably through single-pass generation. Operational environments are dynamic, partially observable, and constrained, and even plausible outputs may require revision after testing against execution results or formal requirements. Iterative correction therefore provides a practical mechanism for improving reliability without assuming that the first output will be correct or safe.

As a result, closed-loop refinement and self-correction are becoming central to credible LLM integration in sensor-driven systems. They shift the focus from static text generation to adaptive interaction with execution, simulation, verification, and feedback, helping align LLM behavior with the demands of real operational environments [12,37,83].

### 4.7. Safety-Aware and Verifiable Architectures

A central requirement for credible LLM integration in sensor-driven systems is that the surrounding architecture must be explicitly safety-aware and verifiable. Unlike conventional software modules designed for narrowly specified tasks, LLMs can generate outputs that are fluent and plausible but incomplete, weakly grounded, or operationally unsafe. Reliable deployment therefore depends not only on model capability, but also on architectural safeguards that constrain, validate, and mediate the LLM’s influence on downstream decisions and actions [36,37,45].

One important design principle is symbolic guidance. In this pattern, LLM reasoning is supported or constrained by symbolic structures such as rules, ontologies, task graphs, logic-based specifications, or explicit operating constraints. This reduces ambiguity and limits unconstrained natural-language generation in domains where ordered procedures and hard constraints are essential [36,37].

A second principle is the use of validation wrappers. LLM outputs are not passed directly to execution layers, but are first filtered through mechanisms such as schema checks, rule-based filters, constraint validators, runtime monitors, or formal verification tools. These wrappers provide a critical boundary between useful semantic assistance and unsafe operational behavior [37,45].

A third principle is fallback policy design. Safety-aware systems must remain robust when the LLM fails, produces uncertain outputs, or generates recommendations that cannot be validated. Fallback policies support graceful degradation, such as reverting to classical controllers, safe modes, human escalation, or simpler rule-based logic [36,37].

A fourth principle is the use of hybrid classical–LLM control structures. In these architectures, the LLM is embedded within a broader control stack in which classical estimation, planning, optimization, and control methods remain responsible for timing-critical and safety-critical functions. The LLM contributes supervisory reasoning and contextual support, while established control mechanisms preserve stability and constraint satisfaction [36,37,82].

These safeguards address a fundamental asymmetry in LLM-enabled systems: the model is useful because it is flexible and generative, but those same properties introduce risk when outputs influence physical behavior without mediation. Architectural safeguards should therefore be treated not as optional add-ons, but as core design elements that determine whether LLM integration is trustworthy in practice [37,86].

Overall, safety-aware and verifiable architectures represent a defining feature of mature LLM integration in sensor-driven systems. They enable semantic reasoning and adaptive assistance while preserving validation, fallback, and controlled execution. Thus, reliable LLM-enabled sensor-driven systems are more likely to emerge from carefully designed hybrid architectures than from attempts to replace classical control structures outright [36,37,45,82].

**Figure 11:**
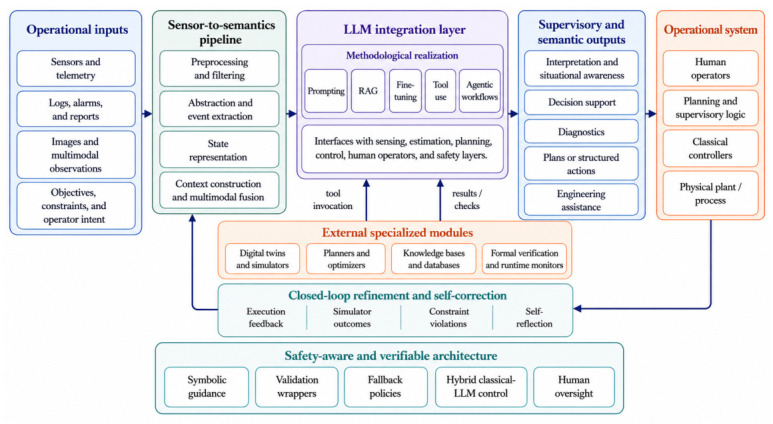
Unified architecture and methodological integration patterns for LLM-enabled sensor-driven control systems.

Figure 11 provides a unified view of the architectural and methodological integration patterns for LLM-enabled sensor-driven control systems. It shows how operational inputs are converted into LLM-usable context through a sensor-to-semantics pipeline, how the LLM layer is realized through prompting, retrieval, fine-tuning, tool use, and agentic workflows, and how its outputs are connected to external modules, supervisory functions, and the operational system. The figure also emphasizes that credible deployment depends on closed-loop refinement, safety-aware validation, fallback policies, and hybrid classical-LLM control structures.

Table 7 further summarizes these integration patterns by comparing their main purpose, typical components, benefits, and implementation challenges. This synthesis highlights that practical LLM deployment in sensor-driven control systems depends not only on model capability but also on careful architectural placement, semantic grounding, legacy-system interoperability, validation mechanisms, and safety-aware integration.

## 5. Representative Application Domains

This section reviews representative application domains in which LLMs are integrated into sensor-driven control systems. Rather than treating the literature as a single body of work, it compares how LLM capabilities are realized across operational settings with different sensing modalities, control requirements, safety constraints, and degrees of human involvement.

Across domains, a consistent pattern emerges: LLMs are most credible as interpretive, supervisory, diagnostic, and human-facing components rather than as replacements for core estimation, control, protection, or actuation mechanisms. The following subsections highlight both recurring integration patterns and domain-specific differences in grounding, placement, and deployment risk.

### 5.1. Robotics and Embodied AI

Robotics and embodied AI constitute one of the most active domains for integrating LLMs into sensor-driven control systems. In this setting, LLMs are mainly used to augment robotic systems with semantic reasoning, task interpretation, multimodal understanding, and context-aware coordination. They are not typically used to replace low-level perception, motion planning, or feedback control, which remain essential for reliable physical execution [87,88,89].

A major application area is task execution. LLMs can translate high-level natural-language goals into structured task plans, decompose objectives into executable subtasks, and support replanning when conditions change. Systems such as SayCan demonstrate how language reasoning can be grounded in robot affordances and skill feasibility, while more recent approaches such as DELTA and ELLMER emphasize long-horizon task decomposition and adaptive execution in dynamic environments [87,89,90].

A second area is manipulation. Robotic manipulation requires object understanding, spatial reasoning, and task context. LLMs can assist by interpreting instructions, inferring affordances and constraints, and linking scene understanding to action sequences. For example, VoxPoser connects language reasoning with vision-based 3D value maps, while ManipLLM and related zero-shot frameworks illustrate the use of multimodal reasoning for flexible object-centric manipulation [91,92,93].

LLMs are also used for multimodal scene understanding and navigation support. Embodied systems often fuse vision, depth, proprioception, language, and other modalities. Architectures such as PaLM-E, RT-2, and ManipLLM show how multimodal representations can connect visual observations, language instructions, and action-relevant reasoning [88,92]. In navigation-oriented tasks, LLMs can help convert high-level directives into route-relevant subgoals and reason about alternatives under uncertainty [87,89].

Overall, robotics demonstrates both the promise and the limitations of LLM-enabled sensor-driven control. LLMs improve flexibility, task interpretation, and semantic coordination, but embodied execution remains constrained by real-time sensing, physical dynamics, uncertainty, and safety. The most credible architectures are therefore hybrid: LLMs support task understanding and supervisory reasoning, while perception modules, planners, and controllers remain responsible for grounded execution [87,89,90,91].

Table 8 summarizes representative uses of LLMs in robotics and embodied AI.

### 5.2. Industrial Automation

Industrial automation is one of the most practically significant domains for LLM integration in sensor-driven control systems. Here, LLMs are not intended to replace PLCs, established control logic, or industrial communication protocols. Their value lies in higher-level semantic interpretation, engineering assistance, maintenance support, and supervisory reasoning across fragmented sources such as sensor streams, alarms, logs, maintenance records, operating procedures, and plant documentation [36,45,94].

First, one important application area is digital-twin-assisted monitoring and decision support. LLMs can interpret digital-twin outputs, summarize plant status, relate predicted states to operating procedures, and support reasoning about deviations, coordination needs, or maintenance implications. In this role, the LLM functions as a semantic layer that translates complex computational outputs into an accessible operational understanding. Recent Industry 5.0 studies further frame LLM–digital-twin integration as a pathway for improving DT construction, operation, cloud–edge collaboration, data analytics, and human-centric industrial decision support [36,82,95]. Digital-twin environments can also provide a structured setting for LLM-guided coordination, learning, and adaptive decision-making [96].

Second, LLMs are relevant to PLC code workflows. Industrial control depends on structured PLC programs, ladder logic, configuration routines, and interface definitions that require careful development and validation. LLMs are increasingly explored as assistants for code generation, explanation, debugging, and verification-oriented engineering support. Their role is to bridge natural-language requirements and technical specifications, while final validation remains with conventional engineering tools and expert review [45,94].

Third, LLMs can support operator-facing industrial robot interaction. In this setting, LLM-based agents can interpret natural-language or speech commands, combine them with visual perception, and invoke dedicated robot-control interfaces for task execution. Recent industrial robotics studies demonstrate this direction in speech- and vision-enabled robotic inspection and quality-control tasks, where LLMs support command interpretation, movement planning, inspection-path generation, and visual queries, while lower-level execution remains handled by dedicated communication interfaces, ROS-based control, or other validated robotic-control layers [97,98].

Fourth, LLMs can assist with maintenance support. They can organize condition-monitoring data, fault histories, alarm sequences, inspection notes, and maintenance records into useful summaries or diagnostic hypotheses. This is valuable when relevant knowledge is distributed across manuals, logs, databases, and expert experience [44].

Finally, LLMs can support supervisory reasoning, including alarm prioritization, procedural interpretation, contingency reasoning, and operator-facing decision support. This placement is especially credible because it keeps LLMs above timing-critical regulation and actuation layers, while established controllers and industrial safety mechanisms remain responsible for execution [36,82].

Overall, industrial automation provides a realistic near-term pathway for LLM deployment. The most defensible architectures are hybrid systems in which LLMs are coupled with digital twins, engineering tools, plant databases, communication interfaces, and supervisory platforms, while PLCs, controllers, and safety systems preserve deterministic and validated execution [36,45,82,97].

### 5.3. Energy and Infrastructure

Energy and infrastructure systems are characterized by large-scale sensing, distributed assets, heterogeneous data streams, and stringent operational constraints. Power grids, substations, renewable-energy facilities, buildings, and critical infrastructure assets generate continuous telemetry, alarms, inspection records, maintenance logs, and planning information. In this context, LLMs are most useful as interpretive and supervisory layers that support decision-making without replacing established estimation, protection, or control mechanisms [99,100,101,102].

One application area is state estimation support. LLMs can contextualize state-estimation outputs, explain uncertainty or anomalous conditions, link measurements to likely operational causes, and translate technical state descriptions into operator-facing summaries [102].

A second area is supervisory grid management. As energy systems incorporate distributed generation, storage, and variable renewable inputs, operators must coordinate many subsystems under changing demand, weather, and equipment conditions. LLMs can help summarize grid status, prioritize alarms, support contingency reasoning, and compare possible responses [103,104,105].

A third area is building and HVAC control support. In HVAC systems, LLMs have been explored as high-level reasoning layers that interpret operational conditions and provide control guidance within MPC-based frameworks, with simulation results indicating potential benefits for energy efficiency and temperature stability [106].

A fourth area is infrastructure monitoring. LLMs can synthesize multimodal monitoring information, summarize inspection findings, contextualize anomalies, and relate current observations to prior incidents, maintenance histories, or procedural knowledge [100,101].

Finally, LLMs can support operational decision support by organizing complex evidence, identifying relevant constraints, comparing candidate responses, and producing concise recommendations for human review [99,101].

Because these systems are safety-critical, geographically distributed, and operationally conservative, fast control, protection, and state-estimation functions should remain under established engineering methods. The most credible architectures therefore couple LLMs with monitoring systems, digital twins, databases, optimization tools, building-management systems, and supervisory platforms, while conventional estimators, controllers, MPC layers, and protection schemes remain responsible for physically grounded operation [99,100,101,106].

### 5.4. Smart Environments and Healthcare

Smart environments and healthcare form a human-centered domain for LLM-enabled sensor-driven control systems. In these settings, sensing is tied not only to physical state monitoring but also to human needs, preferences, activities, and well-being. Smart homes, assisted-living systems, wearable-health platforms, rehabilitation environments, and clinical monitoring systems generate continuous sensor data that must be interpreted in relation to comfort, safety, health status, and user intent. LLMs are therefore most valuable for semantic interpretation, assistive interaction, personalization, and context-aware decision support rather than direct low-level control [101,107,108].

One application area is human-centered sensing. LLMs can transform measurements, alerts, and state estimates into meaningful descriptions of what is happening, why it matters, and which aspects are relevant to users, caregivers, or supervisory systems. This is particularly useful when information is distributed across wearables, cameras, environmental sensors, speech interfaces, and electronic records [107,108].

A second area is assistive interaction. LLMs can serve as natural-language interfaces that explain sensed conditions, answer questions, clarify recommendations, and translate technical outputs into accessible guidance for users, clinicians, or caregivers [101,109].

A third area is user-preference interpretation. Smart and healthcare-oriented systems often need to account for individual habits, comfort preferences, care priorities, and user instructions. LLMs can help interpret these preferences and align system responses with personal needs, especially when preferences are expressed in natural language or change over time [101,110].

A fourth area is context-aware decision support. LLMs can integrate sensor-derived observations with historical context, procedural knowledge, and human-centered priorities to produce recommendations or summaries that are more intelligible than raw signals alone [101,107,108].

At the same time, these environments are safety-sensitive and ethically demanding. LLMs should therefore support interpretation, communication, and personalization while clinical judgment, environmental actuation, and safety-critical enforcement remain under validated sensing, control, and human-oversight mechanisms [101,108,109].

### 5.5. Cross-Domain Diagnosis and Monitoring

A recurring application pattern across sensor-driven control systems is the use of LLMs for diagnosis and monitoring. This pattern appears in industrial automation, energy systems, healthcare, smart environments, and digital-twin-enabled platforms. In these cases, the LLM is not primarily responsible for direct control, but for interpreting system condition, explaining faults, contextualizing anomalies, and supporting maintenance-oriented reasoning [44,111].

One important use is fault analysis. Sensor-driven systems generate large volumes of alarms, logs, exception reports, and condition-monitoring signals, but these observations rarely explain themselves. LLMs can organize heterogeneous evidence into coherent explanations, connect current observations with prior incidents or procedural knowledge, and generate interpretable fault narratives from fragmented technical signals [10,111].

A second use is predictive maintenance. LLMs can synthesize historical records, inspection notes, alarm patterns, and current measurements into maintenance-relevant summaries or recommendations. This helps connect monitoring outputs to maintenance priorities and possible failure trajectories [44].

A third characteristic is the explanation-heavy nature of diagnosis and monitoring. Engineers, operators, clinicians, and supervisors need to understand why a system was flagged, what evidence supports the diagnosis, how confident the interpretation is, and what actions should be considered. LLMs are well suited to this explanatory layer because they can translate technical outputs into accessible language, summarize causal hypotheses, and compare possible interpretations [10,48,111].

This use pattern is transferable because diagnosis and monitoring usually sit above the lowest control layers. LLMs can therefore add value through interpretation and explanation without being entrusted with time-critical actuation. However, outputs must remain traceable to evidence, validated against system data, and clearly separated from final operational judgment, particularly in safety-sensitive settings [48,108,111].

Overall, cross-domain diagnosis and monitoring represent one of the most practical and transferable uses of LLMs in sensor-driven control systems. They show how LLMs can support fault analysis, predictive maintenance, and explanation-rich interpretation while leaving core sensing, estimation, and control mechanisms intact [10,44,108,111].

Table 9 summarizes the major application domains and their most credible LLM roles.

### 5.6. Practical Deployment-Oriented Cases

To further connect the reviewed literature with deployment practice, Table 10 summarizes representative deployment-oriented cases across the main application domains. The table reports the application domain, representative studies, LLM role, sensor or data sources, autonomy level, and evaluation criteria. It is not intended to be exhaustive, but rather to illustrate how LLMs are currently being connected to sensor-driven monitoring, diagnosis, planning, supervision, and constrained runtime support.

## 6. Evaluation Frameworks and Reliability Gaps

Evaluating LLMs in sensor-driven control systems requires more than assessing language quality or isolated task performance. Because these systems combine sensing, state estimation, supervision, human interaction, and sometimes operational decision support, evaluation must consider whether LLM outputs are grounded, timely, safe, useful, and appropriately trusted. This section discusses layered evaluation, operational metrics, control-theoretic considerations for closed-loop operation, reliability bottlenecks, security and trust concerns, and the need for realistic benchmarks.

### 6.1. Layered Evaluation

Evaluation should be layered across the architecture rather than limited to single-output correctness. A useful framework distinguishes three interdependent levels: component-level, system-level, and human-level evaluation. These correspond to grounding accuracy, control relevance, and trust calibration.

At the component level, the central concern is grounding accuracy. Evaluation should determine whether the LLM correctly interprets sensor-derived information, retrieved context, or structured state representations. This includes checking whether summaries, explanations, classifications, or recommendations remain faithful to measurements, logs, images, alarms, or state abstractions. Fluent outputs may still misrepresent sensed conditions, omit critical evidence, or introduce unsupported details [107,111].

At the system level, the focus shifts to control relevance. Even grounded outputs may be operationally weak if they do not support monitoring, diagnosis, planning, maintenance, coordination, or decision-making in the broader system. System-level evaluation therefore asks whether LLM contributions are timely, actionable, appropriately scoped, and compatible with the existing control hierarchy [45,101].

At the human level, evaluation concerns trust calibration. In many sensor-driven environments, LLM outputs are consumed by operators, engineers, clinicians, or supervisors. Evaluation should therefore assess explanation clarity, uncertainty communication, interpretability, and whether users can appropriately decide when to rely on, question, or override the system [37,109].

These layers are interdependent. Weak grounding can undermine system usefulness and distort human trust, while technically correct outputs may still fail if they are irrelevant to operational goals or difficult to integrate into workflows. A layered perspective is therefore essential for evaluating LLM-enabled sensor-driven systems.

### 6.2. Operational Metrics

A credible evaluation framework must be expressed in operational terms. Beyond general language fluency, evaluation should measure whether the system performs reliably under realistic deployment conditions. Key metrics include grounding, robustness, latency, explainability, safety, reliability, and supervisory usefulness [37,45,101].

Grounding measures fidelity to sensor-derived evidence, state representations, retrieved records, or digital-twin outputs. It assesses whether explanations and recommendations are consistent with observed system conditions and traceable to concrete operational data [107,111].

Robustness evaluates whether the system maintains acceptable performance when inputs are noisy, incomplete, delayed, contradictory, or outside expected operating conditions [37,107]. For example, in LLM-based distribution-system state estimation, robustness can be assessed under bad-data and heterogeneous-measurement conditions by comparing estimation errors with conventional and data-driven baselines [112].

Latency measures whether LLM outputs are produced within the temporal requirements of the application, including supervisory decision cycles, sensing frequency, and human or machine response windows [45,101].

Explainability concerns whether users can understand why a recommendation, summary, or diagnosis was produced. This includes rationale clarity, evidence transparency, uncertainty communication, and inspectability [109,111].

Safety examines whether the system respects operational boundaries, avoids unsafe recommendations, and behaves appropriately under abnormal or high-risk conditions [37,45].

Reliability concerns consistency and dependability over repeated operation. A reliable system should produce stable outputs aligned with system requirements across similar inputs and repeated runs [45,101].

Supervisory usefulness evaluates whether the LLM improves monitoring, diagnosis, prioritization, planning, communication, or decision-making in practice [101,109].

Table 11 summarizes these metrics and their relevance to sensor-driven control systems.

Together, these metrics provide a more realistic basis for evaluation than language benchmarks alone. A system that is fluent but weak on grounding, latency, safety, or supervisory usefulness is unlikely to be valuable in operational deployment.

### 6.3. Control-Engineering Implications for Sensor-Driven LLM-Enabled Systems

Because sensor-driven systems often operate in closed-loop settings, the integration of LLMs should be considered not only from an AI or decision-support perspective, but also from a control-engineering perspective. In classical control, closed-loop behavior is evaluated in terms of stability, robustness, transient response, constraint satisfaction, and safety under uncertainty. These requirements become particularly important when LLMs are connected to sensing, planning, supervisory decision-making, or control-code generation modules. Unlike conventional controllers, LLMs do not inherently provide guarantees on stability, boundedness, or robustness. Therefore, their outputs should generally be constrained by model-based controllers, safety filters, supervisory layers, or formally verified interfaces before being allowed to influence physical actuation.

Several reviewed studies indicate that LLMs are most suitable for high-level interpretation, planning, diagnosis, parameter recommendation, and supervisory control, while low-level feedback control should remain within validated control architectures. Model predictive control is especially relevant in this context because it provides an optimization-based framework for handling constraints, multivariable dynamics, and safety requirements [32,47]. Adaptive and learning-based control approaches can further support operation under changing system conditions, but they require careful monitoring to avoid unsafe adaptation or instability [46,70,72]. Latency is another key control concern, since delays introduced by cloud-based inference, tool calls, retrieval, or multi-agent reasoning can degrade closed-loop performance and may destabilize fast dynamical systems. For this reason, LLMs are generally more appropriate for slower supervisory loops unless bounded inference time, fallback mechanisms, and validated low-level controllers are available.

Safety and verification are also central to the deployment and evaluation of LLM-enabled control systems. In safety-critical domains, LLM-generated decisions should be checked against operational constraints, temporal-logic specifications, safety certificates, or rule-based monitors before execution. Recent work on language-guided optimal control, conformal temporal-logic planning, verifiable PLC programming, and LLM-based industrial control agents illustrates the growing interest in combining LLM flexibility with formal or control-theoretic safeguards [45,50,51,94]. Overall, a stronger connection between LLM-based intelligence and classical control principles is necessary to ensure that sensor-driven systems remain stable, robust, safe, and reproducible during real-world closed-loop operation.

### 6.4. Primary Bottlenecks

Despite growing interest in LLMs for sensor-driven control systems, several bottlenecks continue to limit reliable deployment. The most critical are hallucination, weak physical grounding, timing constraints, and the lack of formal stability or correctness guarantees [37,45].

Hallucination remains a central concern. LLMs can generate fluent and plausible outputs that are unsupported, incomplete, or incorrect. In sensor-driven systems, such errors may appear as confident but inaccurate state interpretations, misleading fault summaries, or recommendations without evidential basis [37,111].

Weak physical grounding is equally important. Sensor-driven control systems require accurate interpretation of physical state, constraints, timing, and dynamics. LLMs do not inherently guarantee consistency with physical models or real-time system state. Their outputs are only as reliable as the sensor abstractions, tools, and validation mechanisms that ground them [37,111].

Timing constraints limit deployment in applications with strict response windows. Even when LLMs operate above low-level control loops, outputs may need to support supervisory or operational decisions within bounded time. Retrieval, tool use, simulation, or iterative refinement can further increase latency [45,101].

A further bottleneck is the lack of formal stability or correctness guarantees. Classical estimation, planning, and control methods can often provide guarantees regarding stability, boundedness, feasibility, or correctness under specified assumptions. LLM outputs are probabilistic, context-dependent, and difficult to certify to the same standard [37,45].

These bottlenecks interact. Weak grounding can amplify hallucination; latency can make otherwise correct outputs operationally useless; and the lack of formal guarantees makes it difficult to quantify risk. This explains why the most credible current roles for LLMs remain interpretive, supervisory, diagnostic, and human-facing rather than directly control-executive.

### 6.5. Security and Trust

Beyond performance, LLM integration introduces security and trust concerns. These are especially important because LLMs often sit at the interface between sensing, reasoning, external tools, and human decision-making. Failures may involve adversarial manipulation, misuse of connected tools, unsafe recommendations, or unclear responsibility when harmful outcomes occur [37].

One major concern is prompt injection. LLMs may receive retrieved documents, logs, operator messages, tool outputs, or clinical notes that contain malicious, misleading, or instruction-like content. In sensor-driven systems, external context is often necessary for useful reasoning, but it also creates a channel through which adversarial content can influence model behavior [86].

Mitigating prompt injection requires treating all retrieved or externally supplied content as untrusted data rather than as executable instructions. In industrial environments, this includes alarm logs, historian records, maintenance reports, PLC or SCADA documentation, operator notes, and vendor manuals. In medical sensor environments, it includes electronic health records, clinical notes, wearable-sensor summaries, imaging reports, device logs, and patient-provided text. Practical defenses include separating system-level instructions from retrieved context, enforcing strict prompt templates, sanitizing inputs, source-tagging evidence, and constraining retrieved content to an evidence-providing role. Operational commands, diagnostic suggestions, or control recommendations should be subject to schema validation, engineering or clinical rules, permission controls, safety constraints, human approval, and audit logging before affecting the system [37,86].

A second concern is tool misuse. Tool-augmented architectures allow LLMs to query databases, invoke simulators, analyze records, or interact with external software. While useful, this creates a new security surface. Insufficiently constrained models may call inappropriate tools, request excessive permissions, issue unsuitable commands, or chain tool calls in unintended ways [45].

A third concern is unsafe recommendations. Even when the LLM does not directly actuate the plant or medical device, it may influence maintenance actions, supervisory decisions, clinical interpretation, or operator responses. A recommendation can be dangerous if it ignores constraints, oversimplifies a fault, misrepresents uncertainty, or encourages inappropriate action [37].

A fourth concern is accountability ambiguity. As LLMs become embedded in diagnosis, supervision, planning, and decision support, responsibility can become difficult to assign. If an operator, engineer, or clinician follows an LLM-generated recommendation, or if a failure emerges from interaction between the model and external tools, the boundaries of responsibility may become unclear [37,86].

More broadly, security-aware design principles can mitigate tool misuse, unsafe recommendations, and accountability ambiguity. These include least-privilege tool access, allow-listed tool calls, input and output filtering, retrieval-source authentication, anomaly detection for adversarial or instruction-like content, sandboxed tool execution, and comprehensive logging of prompts, retrieved documents, model outputs, and tool invocations. These mitigations are most effective when combined with human-in-the-loop review, role-based access control, and fallback procedures that keep safety-critical decisions within verified clinical, engineering, or control workflows [37,107,111].

Trust in this context should therefore mean appropriately calibrated reliance based on security, transparency, validation, bounded authority, and resistance to adversarial context manipulation. A trustworthy architecture is one in which the LLM’s role is explicit, permissions are constrained, retrieved content is treated as untrusted evidence, outputs are checked, tool use is monitored, and human or system-level oversight remains clearly defined.

### 6.6. Benchmarking Needs

A major gap in the current literature is the lack of realistic datasets and benchmarks for evaluating LLMs in sensor-driven control systems. Many studies report promising results using narrow tasks, synthetic examples, simplified simulations, or benchmarks that do not capture the complexity of operational environments. As a result, it remains difficult to determine whether reported gains translate into real deployment value [10,37,113].

One limitation is the weak representation of sensing realism. Sensor-driven systems operate on noisy, incomplete, asynchronous, and multimodal data streams, yet many benchmarks provide cleaned inputs, simplified summaries, or static snapshots. Realistic benchmarks should include sensor noise, missing data, conflicting observations, temporal evolution, and multimodal context [10,113].

A second limitation is the limited treatment of operational logic. In real systems, outputs must align with control hierarchies, procedural rules, maintenance workflows, safety constraints, and task dependencies. Benchmarks should therefore test compatibility with supervisory rules, workflow requirements, timing constraints, and domain-specific procedures rather than only linguistic plausibility [10,45].

A third missing element is human oversight. In many systems, LLM outputs are interpreted, validated, and acted on by operators, engineers, clinicians, or supervisors. Evaluation should therefore measure not only correctness, but also whether the system improves user understanding, reduces cognitive burden, supports trust calibration, and helps users make better decisions under uncertainty [55,109].

A fourth limitation is the insufficient representation of deployment constraints. Real environments are shaped by latency, computational limits, access control, privacy requirements, safety rules, and organizational governance. Benchmarks that ignore these constraints may overestimate deployment readiness. More realistic evaluations should include bounded response times, imperfect retrieval, constrained permissions, validation requirements, and fallback procedures [37,101].

To move from general benchmarking needs toward reproducible evaluation, existing domain-specific datasets and simulation environments can be adapted into LLM-oriented benchmark protocols. These resources should not be treated as complete LLM-control benchmarks by themselves, but they provide standardized sensor streams, degradation trajectories, faults, operating conditions, or simulated scenarios from which comparable evaluation tasks can be constructed. In industrial process and SCADA settings, benchmarks such as the Tennessee Eastman Process [114] and SWaT [115] can support evaluation of fault interpretation, alarm explanation, anomaly diagnosis, root-cause reasoning, and safety-aware operator support using process variables, alarms, event logs, and abnormal operating scenarios. In predictive maintenance and PHM, NASA C-MAPSS [116] can be used to assess whether LLMs can interpret multivariate degradation trajectories, explain remaining-useful-life trends, communicate uncertainty, and provide maintenance recommendations without overstating confidence. In autonomous driving and mobile robotics, nuScenes [117] and CARLA-based [118] simulation scenarios can support evaluation of multimodal scene interpretation, risk explanation, high-level planning, and constraint-aware supervisory decision support under realistic sensing conditions.

For reproducibility, such benchmark protocols should clearly specify the LLM input representation, expected output format, scenario or train/test splits, perturbation settings, baseline methods, and reporting metrics. Evaluation should therefore combine domain-specific data or simulation environments with LLM-specific criteria, including grounding fidelity, evidence traceability, robustness to missing or degraded sensor inputs, latency, unsafe recommendation rate, uncertainty communication, consistency across repeated runs, and human- or supervisor-facing usefulness. This would allow future studies to move beyond isolated demonstrations and toward comparable evidence of deployment readiness.

## 7. Future Directions

The future of LLM integration in sensor-driven control systems will depend less on scaling language capability alone and more on improving how these models are grounded, constrained, evaluated, and embedded within operational architectures. Across the reviewed roles and domains, the most credible uses of LLMs augment rather than replace sensing, estimation, planning, and control by adding semantic interpretation, supervisory reasoning, diagnostic support, and human-facing interaction. For practical deployment, these capabilities must also be balanced against latency, memory footprint, privacy requirements, and energy consumption, particularly in resource-constrained sensor and edge-computing environments.

Key research directions include:

- Neuro-symbolic and physics-aware integration: Future systems should connect language-based reasoning with symbolic constraints, physics models, control-theoretic principles, and verified decision structures. This is essential for reducing the gap between fluent language outputs and physically valid behavior.
- Grounded and hybrid architectures: LLMs should be embedded within architectures that use structured state representations, digital twins, tool use, safety wrappers, and classical planning or control components. Such designs allow LLMs to provide semantic flexibility while preserving physical reliability and operational constraints.
- Energy-aware edge and local deployment: Practical deployment will require compact, domain-adapted, and energy-efficient models that can operate under the latency, memory, bandwidth, and power constraints of embedded sensors, edge devices, industrial controllers, wearable systems, healthcare monitors, and infrastructure nodes. Future research should investigate quantization, pruning, distillation, retrieval-efficient architectures, adaptive model selection, caching, edge-cloud partitioning, and event-triggered LLM invocation, where the model is activated only when anomalies, uncertainty, operator queries, or supervisory decisions require semantic reasoning. Evaluation should report computational cost, energy use, inference latency, and communication overhead alongside task performance.
- Human-centered autonomy: Future systems should provide traceable reasoning, audit-ready explanations, calibrated uncertainty, and a clear division of authority between LLMs and human operators. These features are necessary for appropriate trust, accountability, and safe supervisory use.
- Evaluation culture: The field needs benchmarks that go beyond language fluency or isolated task success. Evaluation should reflect sensing realism, operational logic, timing constraints, human oversight, safety requirements, and deployment conditions.

Taken together, these directions indicate that long-term progress will come from deeper integration with the foundations of control engineering rather than attempts to bypass them. The most valuable advances will be systems that are physically grounded, architecturally hybrid, energy-aware, accountable to human oversight, and evaluated under realistic operational conditions.

## 8. Conclusions

This review examined how LLMs are being integrated into sensor-driven control systems, with emphasis on their roles, architectures, application domains, evaluation requirements, and deployment challenges. The main conclusion is that LLMs are most credible when they complement, rather than replace, established sensing, estimation, planning, and control methods. Their strongest near-term value lies in semantic interpretation, contextual reasoning, diagnostic support, operator interaction, engineering assistance, and supervisory decision support. By contrast, direct use of LLMs in fast or safety-critical control loops remains difficult to justify without strong grounding, validation, latency guarantees, and fallback mechanisms.

For practitioners, a conservative deployment pathway is advisable. LLMs should first be introduced in low-risk interpretive roles, such as summarizing alarms, explaining sensor trends, retrieving relevant procedures, assisting with documentation, or supporting fault interpretation. Once these roles are shown to be reliable, LLMs can be extended toward supervisory functions, including maintenance planning, anomaly triage, operator guidance, and what-if analysis. Higher-autonomy roles should only be considered when the system includes structured state representations, verified interfaces, validation wrappers, human approval mechanisms, audit logging, and safe fallback policies. In practical terms, LLMs should remain outside hard real-time control and protection layers unless their outputs are fully constrained and independently verified.

Several design principles follow from this review. LLM-enabled architectures should ground model inputs in sensor-derived evidence, digital twins, control narratives, and domain documentation; restrict tool access according to least-privilege principles; separate retrieved evidence from executable instructions; and check recommendations against physical, operational, clinical, or engineering constraints. Evaluation should also move beyond language fluency and include grounding fidelity, robustness to degraded sensor inputs, latency, unsafe recommendation rate, uncertainty communication, consistency across repeated runs, human usefulness, and computational or energy cost.

Overall, the near-term opportunity for LLMs in sensor-driven control is not the autonomous replacement of classical control but safer and more effective human–machine and system-level decision support. Progress will depend on hybrid architectures that combine the flexibility of language-based reasoning with the reliability of conventional control, estimation, optimization, verification, and safety mechanisms.

## Figures and Tables

**Table 1:** Comparison between this review and related survey publications with respect to the core dimensions emphasized in LLM-enabled sensor-driven control systems.

Focus [Ref.]	Quant. Scope	Sensor-Driven	Role Taxonomy	Architecture	Methods	Domains	Eval./Trust
Autonomous driving [29]	164 refs.; 31 rep. AD studies	✓	∆	∆	✓	∆	∆
Industrial maintenance [8]	140 assessed; 95 synthesized	∆	∆	∆	∆	∆	✓
Robot vision [9]	338 refs.; 5 robot-vision tasks	∆	∆	∆	✓	∆	∆
LLM-based agents [10]	234 refs.; 35 memory models	×	∆	∆	✓	∆	∆
Building energy [11]	54 refs.; 6 applications	∆	∆	∆	✓	∆	✓
Embodied intelligence [12]	105 refs.; 9 datasets tested	∆	∆	∆	∆	∆	∆
Mobile robotics [13]	209 refs.; 5 architectures	∆	∆	✓	✓	∆	∆
Human–robot interaction [16]	122 refs.; 3 HRI modes	∆	∆	∆	∆	∆	∆
Intelligent robotics [30]	170 refs.; 4 robot components	∆	∆	✓	✓	∆	∆
Sensor-driven control (This study)	259 identified; 118 retained	✓	✓	✓	✓	✓	✓

✓: substantially covered in a manner comparable to the scope of this review; ∆: partially covered or covered within a narrower scope; ×: not a central focus. The quantitative-scope column reports the most explicit count available from each survey, including screened or retained records, bibliography size, representative studies, models, tasks, datasets, applications, or system components. These values are descriptive rather than directly comparable, because the reviews differ in methodology, reporting style, and scope.

**Table 2:** Distribution of the 118 reviewed and supporting publications included in the qualitative synthesis.

Classification Dimension	Category	No.
Publication year	Up to 2022: foundational sensor, control, benchmark, and background literature	9
	2023	8
	2024	19
	2025	57
	2026	25
Application domain	Robotics, autonomous systems, and embodied AI	40
	Industrial automation, CPS, digital twins, and fault diagnosis	31
	Energy, buildings, infrastructure, and IoT	18
	Healthcare, wearable, and assistive systems	6
	General LLM/agent, evaluation, and security frameworks	15
	Classical sensor/control background and benchmark resources	8
Type of contribution	Review or perspective paper	33
	Conceptual or architectural framework	16
	Methodological or algorithmic contribution	34
	Application case study or deployed-system study	22
	Experimental or simulation-based demonstration	5
	Evaluation, safety, security, or trustworthiness study	8

Note: Each publication was assigned to one primary category within each classification dimension. The table summarizes the broader reviewed and supporting reference set used in the manuscript, including LLM-focused studies as well as foundational sensor/control, benchmark, and background references.

**Table 3:** Task planning and coordination roles of LLMs in sensor-driven systems.

Function	Supporting Modules/Interfaces	Main Strengths	Main Limitations
Goal decomposition	Scene graphs, symbolic models, digital twins, knowledge graphs [42,52]	Enhances interpretability and bridges human intent to operational tasks	May produce incomplete or infeasible subgoals without strong grounding
Task sequencing	Task planners, process trees, rule engines, workflow managers, automation pyramids [52]	Handles symbolic, procedural, and contextual instructions effectively	Limited guarantees on optimality, timing, and resource efficiency
Replanning and adaptation	Digital twins, runtime monitors, simulators, motion-failure reasoning [55]	Enables semantic adaptation in dynamic operational environments	Sensitive to state quality; prone to error propagation or hallucinations
Multi-agent coordination	Communication protocols, shared memory, multi-agent planners, process trees [61]	Facilitates distributed reasoning across coordinated subsystems	Risk of latency, inconsistency, or coordination ambiguity
Multi-module orchestration	APIs, function calling, digital twins, verification layers, automation pyramids [52,55]	Enables complex tool-mediated workflows in cyber–physical systems	Heavily dependent on interface reliability and external module performance

**Table 4:** Monitoring and diagnostic roles of LLMs in sensor-driven systems.

Function	Supporting Inputs/Modules	Main Strengths	Main Limitations
Anomaly contextualization	Sensor streams, alarms, logs, event histories, knowledge graphs [10]	Improves interpretability and helps distinguish critical faults from transients	Sensitive to input quality; may produce unsupported explanations
Root-cause support	Telemetry trends, maintenance history, procedural knowledge, digital twins [43,65,66]	Organizes complex evidence and supports operator-facing reasoning	Cannot guarantee causal accuracy without strong verification
Fault explanation	Fault alerts, inspection reports, manuals, operator notes [65]	Enhances human understanding in complex scenarios	Risk of oversimplification or omission of uncertainty
Predictive maintenance support	Condition monitoring, failure histories, schedules, spare parts, risk models, digital twins [44,68,69]	Provides integrative reasoning across dispersed maintenance workflows	Heavily dependent on data completeness and quality
Diagnostic workflow coordination	Monitoring platforms, digital twins, tool APIs, verification layers [63]	Supports structured hybrid troubleshooting workflows	Performance strongly tied to external module reliability

**Table 5:** Engineering workflow support roles of LLMs in sensor-driven systems.

Engineering Function	Supporting Inputs/Interfaces	Main Strengths	Main Limitations
Code generation and revision	Generates and revises control logic, Specifications, existing codebases, APIs, PLC tools, verification environments [45,72,73]	Significantly accelerates development and reduces manual coding effort	May produce incorrect, incomplete, or unsafe code without rigorous verification
Commissioning and setup support	Device manuals, wiring diagrams, configuration templates, engineering notes, digital twins [36,74]	Improves efficiency and reduces errors during complex system integration	Configuration errors remain possible if outputs are not thoroughly validated
Documentation and knowledge support	Technical documentation, standards, maintenance records, digital twin data, knowledge bases [44,75]	Makes large volumes of engineering knowledge more accessible	Risk of missing critical details or generating overly confident summaries
Configuration support	Configuration files, interface definitions, system architecture descriptions [36]	Facilitates integration of heterogeneous subsystems	Correctness still depends heavily on human review and testing
Troubleshooting and debugging	System logs, error traces, troubleshooting guides, maintenance history [10,74]	Supports faster and more structured problem diagnosis	May suggest plausible but unverified solutions without strong grounding

**Table 6:** Runtime control participation by LLMs in sensor-driven systems: advantages, risks, and required safeguards.

Runtime Role	Main Advantage	Main Risks	Required Safeguards
Parameter tuning	Enables adaptive adjustment of controller settings in response to changing performance or operating conditions	Poor tuning, instability, performance degradation, or violation of operating limits	Bounded parameter ranges, simulation testing, controller validation, expert approval, and rollback mechanisms
Constrained action recommendation	Supports flexible operational decisions while keeping actions within a predefined safe envelope	Unsafe recommendations, constraint violations, hallucinated justifications, or inappropriate actions under uncertainty	Safety filters, rule-based constraints, optimization checks, runtime monitors, and human or supervisory approval
Execution-time replanning	Improves resilience by adapting plans when faults, anomalies, or environmental changes occur	Infeasible plans, delayed responses, inconsistent task priorities, or unsafe recovery actions	Planner verification, digital-twin testing, feasibility checks, fallback policies, and state-consistency validation
Dynamic task prioritization	Helps allocate attention and resources to the most urgent operational tasks or events	Misprioritization of critical events, delayed mitigation, or conflict with safety procedures	Priority rules, alarm-management logic, operator confirmation, and audit trails
Direct low-level command generation	Offers maximum autonomy and fast semantic-to-action translation in experimental settings	Highest risk: instability, unsafe motion, latency effects, and lack of formal guarantees	Safety shields, formal verification, certified controllers, strict isolation from unrestricted LLM output and emergency stop mechanisms

**Table 7:** Comparative synthesis of architectural and methodological integration patterns for LLMs in sensor-driven control systems.

Pattern	Main Purpose	Typical Components	Main Benefit	Main Challenge
Architectural placement	Define where the LLM sits in the system stack	Interfaces with sensing, estimation, planning, control, operators, and safety layers	Clarifies role and system influence	Strongly affects risk and validation burden
Legacy-system interoperability	Integrate LLMs with existing automation infrastructure	PLCs, SCADA, DCS, BMS, HMIs, historians, middleware, industrial protocols	Enables practical deployment without replacing certified control layers	Requires semantic grounding, access control, latency management, cybersecurity, and auditability
Sensor-to-semantics pipeline	Transform raw sensing into LLM-usable context	State summaries, logs, multimodal abstractions, retrieved context	Improves grounding and interpretability	Poor representations degrade reasoning quality
Methodological realization	Implement LLM capability in practice	Prompting, RAG, fine-tuning, tool use, agentic workflows	Enables flexible task realization	Requires trade-offs among simplicity, cost, and robustness
Tool-augmented and hybrid integration	Combine LLMs with specialized external modules	Simulators, planners, optimizers, digital twins, verification tools	Preserves formal computation and domain grounding	Increases integration complexity and interface dependence
Closed-loop refinement and self-correction	Improve outputs iteratively using feedback	Execution feedback, simulator results, verifier outputs, self-revision	Increases reliability beyond one-shot generation	Adds latency and monitoring complexity
Safety-aware and verifiable architectures	Constrain LLM influence for trustworthy deployment	Symbolic guidance, validation wrappers, fallback policies, hybrid classical–LLM control	Supports bounded and accountable operation	Requires careful architectural design and enforcement

**Table 8:** Representative uses of LLMs in robotics and embodied AI within sensor-driven control systems.

Ref.	Application Focus	Role of the LLM	Sensor/Multimodal Grounding	Main Contribution
[87]	Task execution and embodied decision-making	Translates language goals into feasible robot skill sequences	Robot affordances, skill-value functions, and environmental state	Demonstrates that language reasoning can be constrained by robotic feasibility
[90]	Long-horizon task planning	Decomposes high-level goals into executable subtasks	Task state and planning context	Improves long-horizon planning through LLM-guided decomposition
[89]	Long-horizon embodied execution	Supports adaptive task reasoning under changing conditions	Embodied observations and environmental feedback	Shows extended task completion in dynamic real-world settings
[91]	Robotic manipulation	Interprets instructions and reasons over 3D value maps	Vision-based scene understanding and spatial value representations	Connects free-form language to closed-loop manipulation
[92]	Object-centric manipulation	Provides multimodal reasoning for manipulation tasks	Embodied multimodal observations and object-centric representations	Extends multimodal LLM use toward fine-grained manipulation
[93]	Zero-shot robotic manipulation	Coordinates pretrained models for flexible manipulation	Multimodal robotic observations and manipulation context	Demonstrates a practical zero-shot manipulation framework
[88]	Vision-language-action control	Converts observations and language into action-relevant representations	Visual observations and robot-action data	Transfers large-scale vision-language knowledge to robotic action tasks

**Table 9:** Cross-domain synthesis of representative LLM applications in sensor-driven control systems.

Domain	Credible LLM Roles	Main Integration Pattern	Key Constraint
Robotics and embodied AI	Task interpretation, planning support, scene understanding, manipulation assistance	Hybrid integration with perception modules, planners, affordance models, and controllers	Physical grounding, real-time execution, and safety
Industrial automation	Engineering support, monitoring, maintenance assistance, supervisory reasoning	LLM coupled with digital twins, plant databases, PLC tools, and supervisory systems	Determinism, validation, and industrial safety requirements
Energy and infrastructure	State interpretation, grid supervision, infrastructure monitoring, decision support	LLM coupled with monitoring platforms, optimization tools, digital twins, and databases	Reliability, service continuity, and protection constraints
Smart environments and healthcare	Assistive interaction, preference interpretation, context-aware support, explanation	Human-facing LLM layer connected to validated sensing and supervisory systems	Privacy, ethics, clinical safety, and human accountability
Cross-domain diagnosis and monitoring	Fault explanation, anomaly contextualization, predictive maintenance, evidence synthesis	LLM integrated with diagnostic engines, maintenance databases, and digital twins	Evidence traceability and separation from final operational judgment

**Table 10:** Representative practical deployment cases of LLM-enabled sensor-driven systems.

Application Domain	Studies	LLM Role	Sensor/Data Sources	Autonomy Level	Evaluation Criteria
Autonomous driving and mobile autonomy	[38,39,49]	Scene interpretation, multimodal reasoning, behavior planning, and driving-decision support	Camera, LiDAR, radar, vehicle states, and driving scenes	Supervisory or planning-level support	Scene understanding, planning quality, safety compliance, decision consistency, and task success
Marine and autonomous vessel systems	[40,47]	Mission planning, language-guided control support, and MPC-related decision assistance	Navigation data, vessel states, mission goals, and environmental observations	Supervisory or constrained runtime support	Tracking performance, constraint satisfaction, robustness, safety, and mission completion
Industrial automation and PLC/control systems	[45,52,94]	Control-code assistance, PLC programming, automation-task reasoning, and verification support	PLC variables, SCADA data, process states, alarms, logs, and specifications	Engineering support or bounded supervisory control	Code correctness, verification results, safety-rule compliance, task success, and execution reliability
Smart manufacturing and digital twins	[41,55,96]	Digital-twin interaction, production support, task coordination, and decision assistance	Machine data, digital-twin states, production logs, maintenance records, and operator inputs	Human-in-the-loop or supervisory support	Operational usefulness, coordination quality, response time, productivity, and human acceptance
Fault diagnosis and predictive maintenance	[44,66,71,111]	Fault interpretation, root-cause reasoning, maintenance recommendation, and explanation generation	Sensor measurements, event logs, fault records, equipment states, and knowledge graphs	Advisory or decision-support level	Diagnostic accuracy, explanation quality, evidence grounding, robustness, and maintenance usefulness
Robotics and manipulation	[73,77,98]	Trajectory-level reasoning, task execution support, visual inspection, and control-code generation	Vision inputs, robot states, end-effector trajectories, task instructions, and inspection images	Experimental runtime or constrained supervisory support	Task success, trajectory feasibility, execution safety, perception accuracy, and recovery behavior
Energy systems and smart grids	[80,103,104,112]	Grid monitoring, state-estimation support, dispatch assistance, and multi-agent decision support	SCADA data, grid measurements, smart-meter data, load forecasts, and power-system states	Advisory or supervisory support	Estimation error, robustness to bad data, dispatch quality, security, and operator usefulness
Buildings and home energy management	[11,99,101,106]	Occupant interaction, monitoring, energy-management rule synthesis, and HVAC decision support	Temperature, humidity, occupancy, BMS data, energy use, and user preferences	Advisory or supervisory automation	Energy efficiency, comfort, response time, user satisfaction, and rule validity
Healthcare, wearable, and assistive systems	[28,107,108,109]	Health prediction, elderly-care support, clinical decision support, and assistive interaction	Wearable signals, patient records, clinical notes, robot-interaction data, and monitoring data	Human-in-the-loop decision support	Prediction quality, safety, clinical usefulness, user engagement, and trust calibration
Multi-robot and multi-agent systems	[34,53,61,76]	Task allocation, collaborative planning, target tracking, and multi-agent coordination	Robot states, target observations, communication data, maps, and mission instructions	Supervisory or bounded multi-agent coordination	Mission success, tracking performance, coordination quality, latency, and safety compliance

**Table 11:** Operational evaluation metrics for LLM-enabled sensor-driven control systems.

Metric	Evaluation Focus	Typical Evidence	Main Risk If Neglected
Grounding	Fidelity to sensor-derived evidence and system state	Sensor logs, alarms, state estimates, retrieved records, digital-twin outputs	Hallucinated or unsupported interpretations
Robustness	Performance under noisy, incomplete, delayed, or contradictory inputs	Perturbed sensor data, missing values, degraded scenarios	Fragile behavior under real operating conditions
Latency	Timeliness relative to operational decision cycles	Response time, tool-call time, simulation or retrieval delay	Outputs arrive too late to support action
Explainability	Clarity and inspectability of reasoning and recommendations	Rationales, evidence links, uncertainty statements	Users cannot judge whether to trust the output
Safety	Compliance with constraints and avoidance of unsafe recommendations	Rule checks, safety filters, validation wrappers	Plausible but unsafe operational advice
Reliability	Stability and consistency across repeated use	Repeated trials, similar scenarios, long-duration operation	Inconsistent outputs under similar conditions
Supervisory usefulness	Practical contribution to monitoring, diagnosis, planning, or decision support	Operator studies, workflow outcomes, task performance	Technically correct outputs that do not improve operations

## Data Availability

No new data were created or analyzed in this study. Data sharing is not applicable to this article.
